# Contextualising the Last Survivors: Population Structure of Marine Turtles in the Dominican Republic

**DOI:** 10.1371/journal.pone.0066037

**Published:** 2013-06-19

**Authors:** Carlos Carreras, Brendan J. Godley, Yolanda M. León, Lucy A. Hawkes, Ohiana Revuelta, Juan A. Raga, Jesús Tomás

**Affiliations:** 1 Centre for Ecology and Conservation, University of Exeter, Penryn, United Kingdom; 2 Grupo Jaragua, Santo Domingo, Dominican Republic; 3 Instituto Tecnológico de Santo Domingo, Santo Domingo, Dominican Republic; 4 Cavanilles Institute of Biodiversity and Evolutionary Biology (Scientific Park), University of Valencia, Valencia, Spain; University of Wales Swansea, United Kingdom

## Abstract

Nesting by three species of marine turtles persists in the Dominican Republic, despite historic threats and long-term population decline. We conducted a genetic survey of marine turtles in the Dominican Republic in order to link them with other rookeries around the Caribbean. We sequenced a 740_bp_ fragment of the control region of the mitochondrial DNA of 92 samples from three marine turtle species [hawksbill (n = 48), green (n = 2) and leatherback (n = 42)], and incorporated published data from other nesting populations and foraging grounds. The leatherback turtle (*Dermochelys coriacea*) in the Dominican Republic appeared to be isolated from Awala-Yalimapo, Cayenne, Trinidad and St. Croix but connected with other Caribbean populations. Two distinct nesting populations of hawksbill turtles (*Eremochelys imbricata*) were detected in the Dominican Republic and exhibited interesting patterns of connectivity with other nesting sites and juvenile and adult male foraging aggregations. The green sea turtle (*Chelonia mydas*) has almost been extirpated from the Dominican Republic and limited inference could be made from our samples. Finally, results were compared with Lagrangian drifting buoys and published Lagrangian virtual particles that travelled through the Dominican Republic and Caribbean waters. Conservation implications of sink-source effects or genetic isolation derived from these complex inter-connections are discussed for each species and population.

## Introduction

Many marine vertebrates have complex life cycles requiring the use of different habitats that are often spread over wide spatio-temporal scales and usually result in a network of connections among different populations and between populations and distant feeding grounds [1]–[7]. This complex structure is especially relevant to endangered species, in which threat may be highly localized, yet have a potentially profound effect on distant areas. Thus, the knowledge of migratory pathways, population structure and connectivity of the most threatened populations in comparison to other populations and feeding grounds is crucial for an effective application of conservation actions. Marine turtles are one of the best examples of this complex structuring [8]–[9] and highly migratory behaviour [10], and have been subject to increasing focus for molecular research and conservation over the last few decades. This is especially relevant for management, as all marine turtle species are of conservation concern, and action plans often need to be international in scope [11]. As the Caribbean hosts numerous populations of several marine turtle species [12], gaps in information may lead to undiagnosed population sinks to otherwise protected stocks [11], [13].

The Dominican Republic has, for many years, presented a gap of knowledge in marine turtle biology and conservation. Although it has been suggested as an important nesting area for several marine turtle species [14]–[15] there has been a paucity of monitoring data. Furthermore, there is clear evidence of a long history of harvesting and exploitation of marine turtle meat, eggs and shell as an important resource for local communities [14]–[18]. In addition to these threats, turtles are also incidentally captured at sea [19]–[20]. Although marine turtles have been legally protected by law since 1966 in the Dominican Republic [21]–[22] it was estimated that there was an annual capture of between 1000 and 2000 green (*Chelonia mydas*), loggerhead (*Caretta caretta*) and leatherback (*Dermochelys coriacea*) turtles during the 1980s [19]. In addition, a total of 4366 kg of hawksbill (*Eretmochelys imbricata*) shell was exported to Japan between 1970 and 1986 [23]. Turtle shell exploitation was also detected more recently [17]–[18], [24]–[25]. The country also receives four million tourists annually that result in a significant degradation of coastal habitats [26]–[27].

The consequences of the accumulation of these threats to the Dominican Republic nesting populations were not properly addressed until a recent study [22], which suggested that the Dominican Republic is an important nesting area for the hawksbill and leatherback turtle and includes some sporadic nesting by green turtles. However, the persistence of threats appears to have led to population reduction and a significant contraction of nesting habitats, with nesting largely restricted to protected areas [14]–[15].

Genetic markers have been widely used to establish links between populations and feeding grounds and to infer relative exposure to threats [8]–[9], [28]. Maternally inherited mitochondrial DNA (mtDNA) is particularly well suited to assess such links as nesting female turtles exhibit marked site fidelity [8]–[9] permitting the definition of isolated Management Units (MUs) [29]. Juvenile turtles, however, may be widely dispersed and occupy foraging areas comprised of turtles of differing provenance [28], [30]–[31]. For some species it has been shown that this mixing still remains after sexual maturity and could lead to male mediated gene flow between populations [32]–[33] although not in all cases [34]. For this reason, the concept of Regional Management Units (RMUs) has been proposed [13]. These would function as higher level conservation units that include all isolated populations (MUs) linked by the use of common feeding grounds, since mortality occurring in shared feeding areas will affect contributing nesting populations [13]. Mixed Stock Analysis (MSA) has provided the tool with which to link feeding grounds with nesting habitats and hence establish relationships at the RMU level for many sea turtle species testing what proportion of turtles from a feeding ground come from each nesting population from a *mixed stock* point of view [30], [35]–[42]. The recent development of the ‘many to many’ analysis [43] allowed the testing of how many individuals from a nesting population use each feeding ground from a *nesting population* point of view [44]–[45].

Other approaches have involved the study of ocean currents, and have demonstrated that they may play a crucial role in defining hatchling and juvenile marine turtle dispersion [30], [41], [46]–[48]. To understand population connectivity, therefore, empirical data describing sea surface currents may be informative [49]. For example, data describing the tracks of Lagrangian drifting buoys [44], [50], or biophysical modelling of oceanic dispersal [51]–[53] could be used to simulate the movements of a passively drifting hatchling turtle to complement the tagging of individuals [51], [54]–[56]. Thus, a multidisciplinary approach has been recommended to assess both the connectivity among populations [57] and the definition of RMUs [13].

Here we combine genetic data from the Dominican Republic turtle nesting population with published genetic data from other nesting populations and feeding grounds, as well as tracks of passive drifter buoys and satellite tracking of adult turtles to: 1) determine if Dominican Republic turtle nesting populations are isolated from others in the region; 2) determine if mtDNA sequences reflect population size reductions; and 3) determine which feeding grounds are used by Dominican Republic marine turtles.

## Materials and Methods

**Figure 1:**
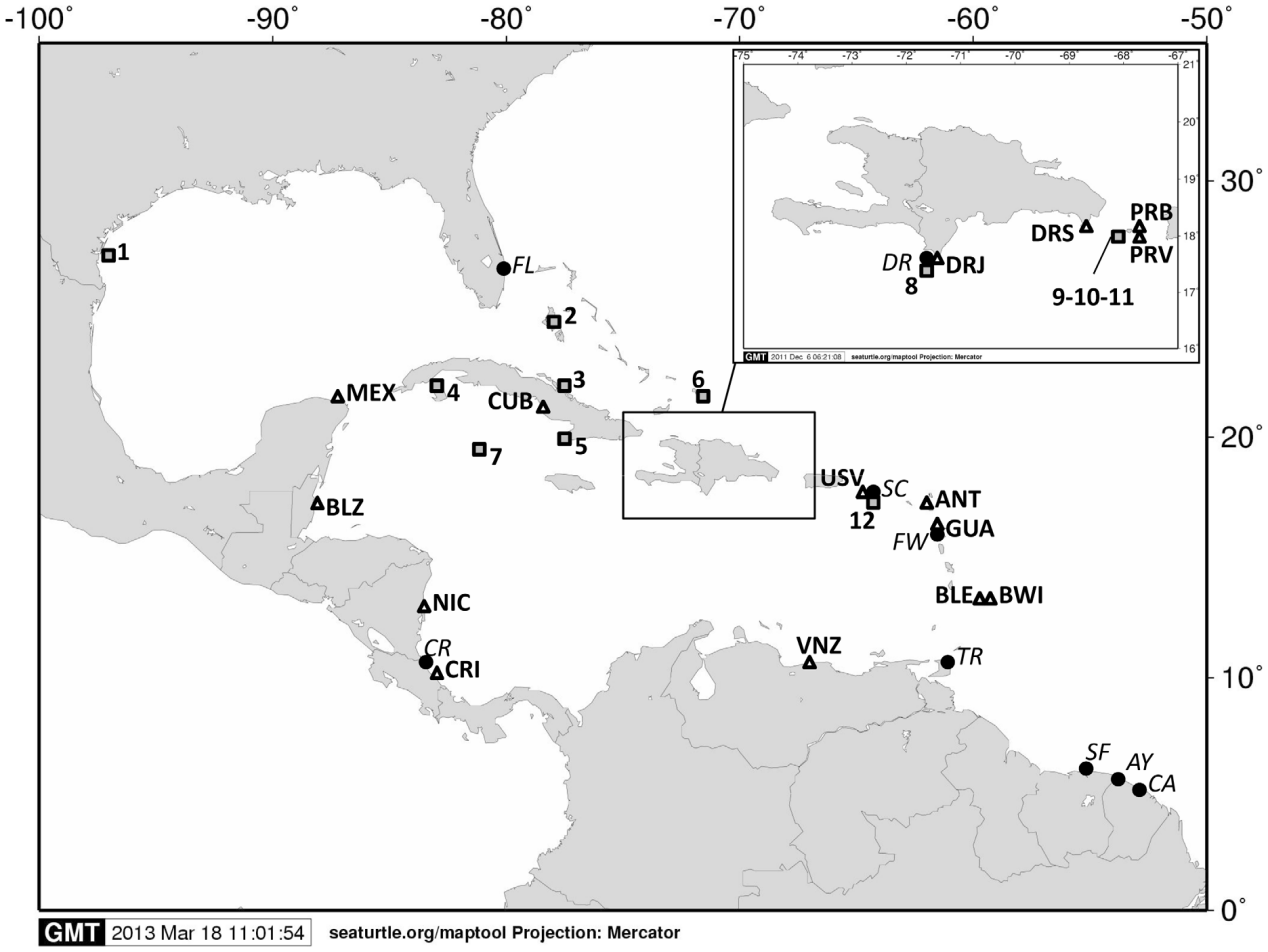
Marine turtle nesting sites and feeding grounds considered in this study. Leatherback nesting sites are represented by black circles labelled in italics. AY: Awala-Yalimapo; CA: Cayenne; CR Costa Rica (Atlantic); FL: Florida; FW: French West Indies; SC: Saint Croix; SF: Suriname/FG; TR: Trinidad; DR: Dominican Republic. Hawksbill nesting sites are represented by white triangles labelled in bold. ANT: Antigua; BLE: Barbados Leeward; BWI: Barbados Windward; BLZ: Belize; CRI: Costa Rica; CUB: Cuba; GUA: Guadeloupe; MEX: Mexico; NIC: Nicaragua; PRV: Puerto Rico [35]; PRB: Puerto Rico [64]; USV: US Virgin Islands; VNZ: Venezuela; DRJ: Dominican Republic-Jaragua; DRS: Dominican Republic-Saona. The hawksbill population of BRZ: Brazil and the leatherback populations of BZ: Brazil and SA: South Africa are not included in the map. Hawksbill feeding grounds are represented by numbered grey squares. 1. Texas; 2. Bahamas; 3. Cuba D; 4. Cuba B; 5. Cuba A; 6. Turk and Caicos; 7. Caiman Islands; 8. Dominican Republic; 9. Puerto Rico residents; 10. Puerto Rico recruits; 11. Puerto Rico pooled; 12. US Virgin Islands. Sampling sites of the present study were the Jaragua National Park (DRJ or DR) and Saona Island (DRS). Map created using MAPTOOL [106].

During the 2007 and 2008 nesting seasons (from March to October), samples from three nesting marine turtle species were collected within an extensive monitoring project to assess nesting marine turtle abundance and distribution in the Dominican Republic [22] (Figure 1, Table 1). Samples from green turtles (n = 2; DRS), hawksbill turtles (n = 48; DRS n = 33, DRJ n = 15) and leatherback turtles (n = 42; DRJ) were collected from the Jaragua National Park (DRJ/DR) and Saona Island (DRS), with the permission and support of the office of Protected Areas of the Ministry of Environment and Natural Resources of the Dominican Republic Government. Muscle and skin samples were collected from dead hatchlings found in nests after emergence, so sampling had no impact on living animals and thus the study did not require the approbation of any ethical animal committee. Only one hatchling per nest and female was sampled in order to ensure independence of samples. Due to the lack of resources in the study area and the low density of nesting events in some places and species, it was not possible to identify and tag all females while nesting. However, a filtering method applied in other marine turtle species that combine remigration interval, sample location and haplotype found [58] was applied in order to avoid the risk of pseudoreplication. Samples were stored in 95% ethanol (n = 1 hatchling sample per nest). Finally, samples were transported for analysis following international CITES regulations.

**Table 1:** Haplotype frequencies found in Dominican Republic marine turtle populations.

Hawksbill (*E. imbricata*)				Leatherback (*D. coriacea*)			Green (*C. mydas)*		
Haplotype	GAN	DRJ	DRS	Haplotype	GAN	DR	Haplotype	GAN	DRS
Ei-A01 (A/CU1)	EF210779	1	3	Dc_A1 (A)	EF513272	38	CM-A5	Z50127	2
Ei-A09 (F/c)	EF210783	2	–	Dc_C (C)	EF513272	4			
Ei-A11 (F/PR1)	EF210784	3	22						
Ei-A18 (L/PR3)	EF210786	–	2						
Ei-A20 (N/PR2)	EF210788	–	6						
Ei-A23 (Q/MX1)	EF210791	4	–						
Ei-A43 (Q/MX2)	EF210794	4	–						
Ei-A47 (L/PR3)	EF210787	1	–						
**TOTAL**		**9**	**30**			**42**			**2**

Hawksbill frequencies are given for 740_bp_ haplotypes and the equivalences for 380_bp_ and 480_bp_ haplotypes are given in brackets respectively (380_bp_/480_bp_). Leatherback frequencies are given for 711_bp_ haplotypes and the equivalences for 496_bp_ are given in brackets. DRJ/DR: Jaragua National Park; DRS: Saona Island. GAN: GenBank Accession Number of each haplotype. Hawksbill haplotypes defined in [35]. Leatherback haplotypes defined in [62]. Green haplotype described in [84] and compiled in http://accstr.ufl.edu/.

DNA was extracted using the QIAamp extraction kit (QIAGEN®) following the manufacturer’s instructions. We amplified a ∼740_bp_ fragment of the mtDNA control region using the primers LCM15382 (5′-GCTTAACCCTAAAGCATTGG-3′) and H950 (5′GTCTCGGATTTAGGGGTTT-3′) [59]. This fragment includes the region historically surveyed for several marine turtle species in previous studies having lengths of 496_bp_ (leatherback sea turtle), 491_bp_ (green sea turtle) or 384_bp_/480_bp_ (hawksbill sea turtle). Our 25 µL polymerase chain reaction (PCR) included the following: genomic DNA, 1× PCR Buffer, 2 mM MgCl_2_, 0.12 mM dNTP, 0.2, µm of each primer and 0.04 U/µL of Taq polymerase. After an initial 5 min denaturing step (94°C), our PCR protocol consisted of 35 cycles of the following temperature regime: 1 min at 94°C (denaturing), 1 min at 52°C (annealing) and 90 s at 72°C (extension). In addition, we included a final extension step of 10 min at 72°C. Following PCR, we removed single-stranded DNA by digesting 5 µL of PCR product with 2 µL of a combined Exonuclease I and Shrimp Alkaline Phosphatase solution (ExoSAP-IT®). The reaction mixture was incubated for 15 min at 37°C, followed by other 15 min incubation at 80°C to inactivate the two enzymes. We sequenced both forward and reverse strands using the BigDyeTM Primer Cycle Sequencing Kit (Applied Biosystems) run on an automated DNA sequencer (ABI PRISM 3100). For each sequencing reaction, we used 2 µL of our PCR product in a 10 µL reaction mix under the following conditions: 1 m denaturing step at 96^a^ followed by 25 cycles consisting of an initial denaturing of 10 s at 96°C, 5 s at 50°C (annealing) and 4 m at 60°C (extension). Products were purified by ethanol precipitation before enter the sequencer.

Sequences were aligned by eye using the program BioEdit 5.0.9 [60] and compared with the short (<500_bp_) haplotypes previously described for the leatherback turtle [61]–[63] and the hawksbill turtle [35], [38], [40], [64]–[67]. Additionally, the whole fragment was compared to known long (>500_bp_) haplotypes described in those manuscripts that used the same or similar primers in the leatherback turtle [62]–[63] and the hawksbill turtle [35], [40]–[42], [67]. Green turtle sequences were compared with the haplotypes found in the database maintained by the Archie Carr Center for Sea Turtle Research (http://accstr.ufl.edu/) that includes all published haplotypes. Posterior statistical analyses were carried out with short, long sequences or both depending on the data found in the published literature.

### Population Structure

Differences in haplotype frequencies of samples from the same species at different locations within Dominican Republic were assessed using a Chi-square test. Values were compared to the distributions observed by randomizing individuals among populations using Monte-Carlo resampling [68] as implemented in the program CHIRXC [69]. Additionally we computed the exact test based on haplotypes frequencies [70] in Arlequin 3.0. Both analyses were used to test if samples from different locations could be grouped or should be considered separately.

In order to assess the genetic diversity compared to the other Atlantic populations we calculated haplotype diversity (*h*) and nucleotide diversity (π) [71] of each population and species using the program Arlequin 3.0 [72]. Fu’s Fs neutrality test for the detection of population growth [73] was undertaken with the DnaSP 5.0 software package [74] for each nesting population of the Dominican Republic. Fs tends to be negative under an excess of recent mutations and a significant negative value was taken as evidence of recent population expansion. Differentiation among population pairs was assessed considering frequency based genetic distances (Φ_st_) using Arlequin 3.0 [72]. Significance of differentiation was tested using a Chi-square test and computing the exact test based on haplotypes frequencies as explained above. Genetic distances were used to perform a Principal Coordinate Analysis (PCA) with the package GenAlEX 6.2 [75] in order to distribute, in a two-dimension space, the genetic variability found. A sequential Bonferroni correction was not applied for multiple pair-wise comparisons, since they dramatically increase the probability for type II error (β: assume no differentiation when it does exist), an effect that becomes worse as many P-values are discarded [76]–[77]. In substitution, we applied the False Discovered Rate (FDR) correction that calculates the most appropriate threshold for the P-value significance considering the multiple comparisons involved in the analysis under an expected original threshold of P<0.05 [78].

### Hawksbill Juvenile and Male Dispersion

Considering our samples and published information available, a mixed stock analysis was performed in order to test the dispersion of hawksbill juveniles originating in Dominican Republic nesting areas. We used the *rookery centred* approach of the ‘many-to-many’ analysis [43] to test how juveniles hatched in the Dominican Republic disperse to all known Caribbean feeding grounds and a *mixed stock centred* approach to test the relative importance of Dominican Republic to the individual feeding grounds. For the nesting population baseline we used the haplotype frequencies previously described in the literature [35], [40], [67] but incorporating the frequencies of the Dominican Republic Jaragua and Saona (*present study*) (Table S1). Additionally, we tested the contribution of the Caribbean nesting populations to the juvenile feeding ground located south-west of the Dominican Republic [38]. All ‘many to many’ mixed stock analyses were conducted using only the short (380_bp_) haplotype frequencies, as this analysis requires detailed information of haplotype frequencies of nesting populations and feeding areas and the dataset regarding putative feeding grounds available in the literature is most extensive for this fragment (Table S2). Nesting populations and feeding grounds located in the eastern Atlantic [79]–[80] were not included in the analysis as they have been shown to be highly isolated from all Caribbean populations and their contribution to Caribbean feeding grounds has been shown to be negligible [79]. Finally, a ‘one-to-many’ analysis was conducted to test the possible contribution of Dominican Republic turtles to the adult male aggregation found in Puerto Rico [35]. This analysis was conducted using either short (Table S1) or long sequences (Table S3). As we compared the nesting population haplotype frequencies to a single foraging area, the haplotype frequencies of other feeding grounds were not needed. All mixed stock analysis included population size as a weighting factor as several studies have proved that the inclusion of this factor improved the accuracy of results [41], [81]. Source population sizes (as mean number of nesting females) were taken from the literature [22], [67], [82] (Tables S1 and S3).

### Lagrangian Buoys Dispersion

In order to simulate hatchling turtle dispersal, data describing the tracks of satellite-tracked surface drifter buoys were obtained from the Global Drifter Program of the National Oceanic and Atmospheric Administration (NOAA, USA). These Lagrangian buoys are periodically released throughout the year at varying locations and are tracked by satellite (RAMS, Argos, EOLE), providing several positional fixes per day (accuracy 0.1–2.0 km; http://www.aoml.noaa.gov/). An initial query within the locations database was undertaken in order to select all locations plotted near (<50 km) either the Jaragua National Park (DRJ/DR) or Saona Island (DRS) nesting populations. Then, all buoys with at least one location obtained near one of these Dominican Republic nesting areas were selected as an evidence of passive arrival or departure from the area.

## Results

The two green turtle samples were collected in Saona Island (DRS) and exhibited the haplotype CM-A5 (Table 1) which is found at high frequency in Suriname [83], Aves Island and Venezuela [84] but is also present at very low frequencies in other nesting populations of the Atlantic, including Mexico [83], Costa Rica [85], and Sâo Tomé and Príncipe [86]. Due to this low sample size, no further statistical analyses were performed for this species.

### Leatherback Population Structure

**Figure 2:**
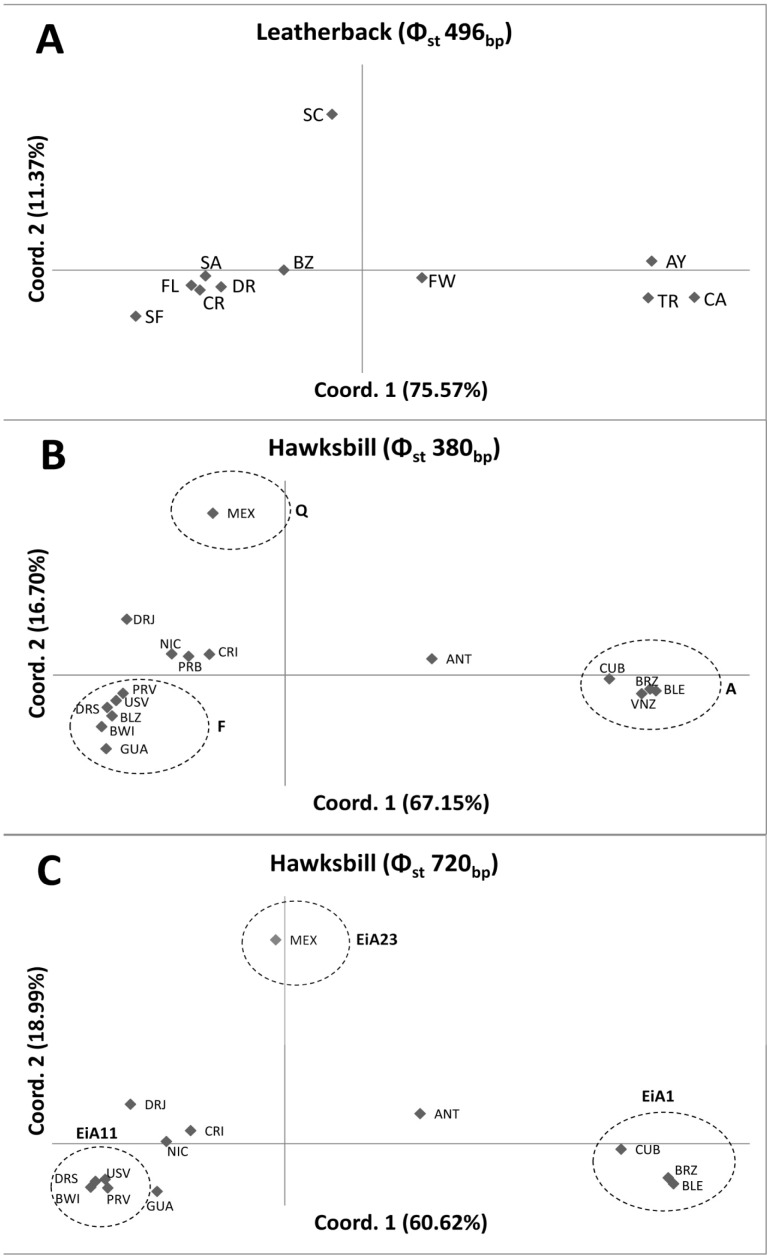
PCA including Atlantic leatherback and hawksbill populations using Φ_st_. The percentage of the variability explained by each coordinate is shown in brackets. For the leatherback turtle(A) PCA was done using the 496bp fragment. For the hawksbill turtles PCA was done either considering the 380_bp_ fragment (B) and the 720_bp_ fragment (C). Leatherback nesting populations: AY: Awala-Yalimapo; BZ: Brazil; CA: Cayenne; CR Costa Rica (Atlanic); FL: Florida; FW: French West Indies; SA: South Africa; SC: St. Croix; SF: Suriname/FG; TR: Trinidad; DR: Dominican Republic. Hawksbill nesting populations: ANT: Antigua; BLE: Barbados Leeward; BWI: Barbados Windward; BLZ: Belize; BRZ: Brazil; CRI: Costa Rica; CUB: Cuba; GUA: Guadeloupe; MEX: Mexico; NIC: Nicaragua; PRV: Puerto Rico [35]; PRB: Puerto Rico [64]; USV: US Virgin Islands; VNZ: Venezuela; DRJ: Dominican Republic-Jaragua; DRS: Dominican Republic-Saona. Dashed circles represent the groups detected in previous studies [40] indicating the haplotype found at higher frequency.

Two haplotypes were found among leatherback samples when considering either the 496_bp_ fragment or the 711_bp_ fragment (Table 1). Genetic variability of the Dominican Republic nesting population was similar or higher than the other Atlantic populations [DR: *h* (SD) = 0.176 (0.074), π (SD) = 0.0014 (0.0012)] with the exceptions of some populations outside the Caribbean like Awala-Yalimapo [AY: *h* (SD) = 0.780 (0.061), π (SD) = 0.0051 (0.0033)], Cayenne [CA: *h* (SD) = 0.519 (0.030), π (SD) = 0.0042 (0.0027)], French West Indies [FW: *h* (SD) = 0.340 (0.090), π (SD) = 0.0027 (0.0020)], Saint Croix [SC: *h* (SD) = 0.589 (0.067), π (SD) = 0.0024 (0.0018)] and Trinidad [TR: *h* (SD) = 0.501 (0.043), π (SD) = 0.0040 (0.0026)]. No recent expansion was suggested for Dominican Republic leatherback population, independently of the length of the marker used (Fu’s Fs neutrality test: 496_bp_: Fs = 2.664, P = 0.198; 711_bp_: Fs = 3.307, P = 0.134). As previous studies with the 496_bp_ fragment indicated [61], pairwise population analysis showed very deep differentiation between all Atlantic/Indic and all Pacific populations (data not shown) so the latter group of populations were not considered for future analysis. The Dominican Republic nesting population exhibited moderate levels of differentiation with other Atlantic/Indian ocean nesting populations (Table 2) being significantly different from Awala-Yalimapo, Cayenne, Saint Croix and Trinidad. The separation of these four populations from the others was corroborated by the PCA analysis, with their two principal coordinates explaining an accumulated 86.9% of the genetic variability found in all populations (Figure 2A). The comparisons using the 711_bp_ fragment yielded similar results (Table 2).

**Table 2:** Genetic distances (Φ_st_) between Atlantic/Indic leatherback nesting populations.

	AY	BZ	CA	CR	FL	FW	SA	SC	SF	TR	DR	Reference
AY	–	0.168*	0.037*	N	N	0.108*	N	N	N	N	0.277*	[63]
BZ	0.184*	–	0.193	N	N	−0.019	N	N	N	N	−0.061	[62]
CA	0.065*	0.202	–	N	N	0.095	N	N	N	N	0.285*	[63]
CR	0.301*	−0.065	0.306*	–	N	N	N	N	N	N	N	[61]
FL	0.299*	−0.009	0.331	−0.019	–	N	N	N	N	N	N	[61]
FW	0.126*	−0.018	0.097	0.040	0.090	–	N	N	N	N	0.022	[63]
SA	**0.268**	−0.032	0.308	−0.035	0.000	0.072	–	N	N	N	N	[61]
SC	0.251*	0.057	0.265*	0.108*	0.113	0.095*	0.093	–	N	N	N	[61]
SF	0.406*	0.058	0.415*	0.020	0.000	0.145	0.000	0.180*	–	N	N	[61]
TR	0.061*	0.152	−0.038	0.243*	0.274	0.057	0.253	0.222*	0.346*	–	N	[61]
DR	0.292*	−0.061	0.289*	−0.027	0.001	0.022	−0.014	0.106*	0.037	0.226*	–	PS

Genetic distances based on 496_bp_ and 711_bp_ sequences. Significant values given by the exact test and after FDR correction are marked with (*) for the exact text and in bold for the Chi-square test (for a P<0.05 FDR_496bp_ = 0.0109). AY: Awala-Yalimapo; BZ: Brazil; CA: Cayenne; CR Costa Rica (Atlanic); FL: Florida; FW: French West Indies; SA: South Africa; SC: St. Croix; SF: Suriname/FG; TR: Trinidad; DR: Dominican Republic. Pacific populations [61] were included in the analysis but have not been included in the table as all were significantly different from all Atlantic/Indic populations. N: pairwise comparison not possible as 711_bp_ sequences were not available for some of the populations. PS: Present Study.

### Hawksbill Population Structure

A total of 8 haplotypes were found in the Dominican Republic hawksbill samples using the whole 720_bp_ sequence (Table 1). When we truncated the sequence for comparisons with previous studies, we found 5 haplotypes considering the 380_bp_ and 6 considering the 480_bp_ fragment (Table 1). The two sample sites in the Dominican Republic were genetically different considering both short and long fragments (380_bp_ fragment: Φ_st_ = 0.123; 720_bp_ fragment: Φ_st_ = 0.223; P<0.001 in all cases, both for the Chi-square and exact test) and hence were treated as two independent units for all posterior analysis. Genetic variability of the two Dominican Republic populations was similar or higher than other nesting populations [380_bp_: DRJ: *h* (SD) = 0.638 (0.093), π (SD) = 0.0037 (0.0027); DRS: *h* (SD) = 0.526 (0.089), π (SD) = 0.0044 (0.0029); 720_bp_: DRJ: *h* (SD) = 0.848 (0.054), π (SD) = 0.0047 (0.0029); DRS: *h* (SD) = 0.527 (0.089), π (SD) = 0.0035 (0.0021)]. No recent expansion was suggested for either populations irrespective of the length of the marker used (Fu’s Fs neutrality test: 380_bp_: DRJ: Fs = 0.440, P = 0.269; DRS: Fs = 1.957, P = 0.167; 720_bp_: DRJ: Fs = 0.286, P = 0.233; DRS: Fs = 3.581, P = 0.062). Analysis of genetic structuring of Dominican Republic nesting beaches in relation to other populations in the Caribbean showed deep levels of differentiation with the exceptions of the DRS population that showed no significant differences to the proximate nesting aggregation of Mona Island in Puerto Rico (PRV), the populations of Belize (BLZ), Barbados Windward (BWI) and U.S. Virgin Islands (USV) (Table 3). The similarity between DRS and PRV, and between DRS and USV was confirmed also for the 720_bp_ fragment while BWI yielded significant differentiation with DRS by means of the exact test, but not for the Chi-square test (Table 3). The lack of differentiation between DRS and BLZ was not confirmed as no long sequences were available for the latter. The accumulated first two coordinates of the PCA explained a high percentage of the genetic variability found both for the 380_bp_ (83.9%; Figure 2B) and the 720_bp_ (79.61%; Figure 2C) fragments and confirmed the complete isolation of DRJ and the relative proximity of DRS to PRV, USV and BWI (Figure 2C).

**Table 3:** Genetic distances (Φ_st_) between Caribbean hawksbill nesting populations.

	ANT	BLE	BWI	BLZ	BRZ	CRI	CUB	GUA	MEX	NIC	PRV	PRB	USV	VNZ	DRJ	DRS	*Reference*
ANT	**–**	0.373*	0.407*	N	0.381*	0.203*	0.206*	0.563*	0.514*	0.284*	0.495*	N	0.414*	N	0.345*	0.418*	[67]
BLE	0.372*	**–**	0.913*	N	0.076*	0.661*	**0.076**	0.951*	0.978*	0.678*	0.854*	N	0.849*	N	0.912*	0.902*	[40]
BWI	0.460*	0.925*	**–**	N	0.896*	0.070*	0.740*	0.287*	0.679*	0.032*	0.041*	N	−0.012	N	0.132*	0.014*	[40]
BLZ	0.381*	0.916*	−0.027	**–**	N	N	N	N	N	N	N	N	N	N	N	N	[107]
BRZ	0.380*	0.099*	0.906*	0.892*	**–**	0.667*	0.085*	0.936*	0.942*	0.684*	0.851*	N	0.843*	N	0.886*	0.888*	[67]
CRI	0.231*	0.644*	0.085*	0.025	0.651*	**–**	0.517*	0.279*	0.412*	0.010*	0.134*	N	0.070*	N	0.064*	0.078*	[67]
CUB	0.210*	0.078	0.762*	0.716*	0.090*	0.511*	**–**	0.824*	0.758*	0.568*	0.755*	N	0.720*	N	0.688*	0.740*	[67]
GUA	0.599*	0.950*	0.008	0.070	0.937*	0.203*	0.839*	**–**	0.811*	0.240*	0.347*	N	0.308*	N	0.415*	0.405*	[67]
MEX	0.618*	0.997*	0.636*	0.629*	0.973*	0.311*	0.850*	0.723*	**–**	**0.420**	**0.649**	N	**0.613**	N	**0.438**	0.661*	[35], [67]
NIC	0.329*	0.707*	0.048*	0.012*	0.711*	0.005*	0.594*	0.136*	0.270*	**–**	0.085*	N	0.030*	N	0.055*	0.040*	[67]
PRV	0.519*	0.840*	0.045*	0.048*	0.838*	0.138*	0.749*	0.081*	0.443*	0.111*	**–**	N	0.025*	N	0.194*	−0.012	[35]
PRB	0.316*	0.848*	0.212*	0.111*	0.828*	0.043*	0.625*	0.428*	0.536*	0.093*	0.167*	**–**	N	N	N	N	[48]
USV	0.453*	0.852*	−0.019	−0.023	0.846*	0.078*	0.730*	0.035*	0.466*	0.044*	0.036*	0.182*	**–**	N	0.132*	−0.004	[67]
VNZ	0.231	0.000	0.838*	0.753*	−0.003	0.495*	−0.017	0.923*	0.994*	0.612*	0.786*	0.611*	0.773*	**–**	N	N	[38]
DRJ	0.433*	0.948*	0.165*	0.102*	0.919*	0.079*	0.742*	0.343*	0.281*	0.053*	0.157*	0.114*	0.127*	0.842*	**–**	0.136*	PS
DRS	0.461*	0.901*	0.010	0.005	0.887*	0.085*	0.750*	0.068*	0.552*	0.064*	−0.014	0.131*	0.002	0.800*	0.123*	**–**	PS

Below the diagonal distances based on 380_bp_ traditional sequence, above diagonal distances based on the 720_bp_ sequence. Significant values given by the exact test and after FDR correction are marked with (*) for the exact text and in bold for the Chi-square test (for a P<0.05 FDR_380bp_ = 0.0093; FDR_740bp_ = 0.0101). ANT: Antigua; BLE: Barbados Leeward; BWI: Barbados Windward; BLZ: Belize; BRZ: Brazil; CRI: Costa Rica; CUB: Cuba; GUA: Guadeloupe; MEX: Mexico; NIC: Nicaragua; PRV: Puerto Rico [35]; PRB: Puerto Rico [64]; USV: US Virgin Islands; VNZ: Venezuela; DRJ: Dominican Republic-Jaragua; DRS: Dominican Republic-Saona. N: pairwise comparison not possible as 720_bp_ sequences were not available for some of the populations. PS: Present Study.

### Hawksbill Juvenile and Male Dispersion

**Figure 3:**
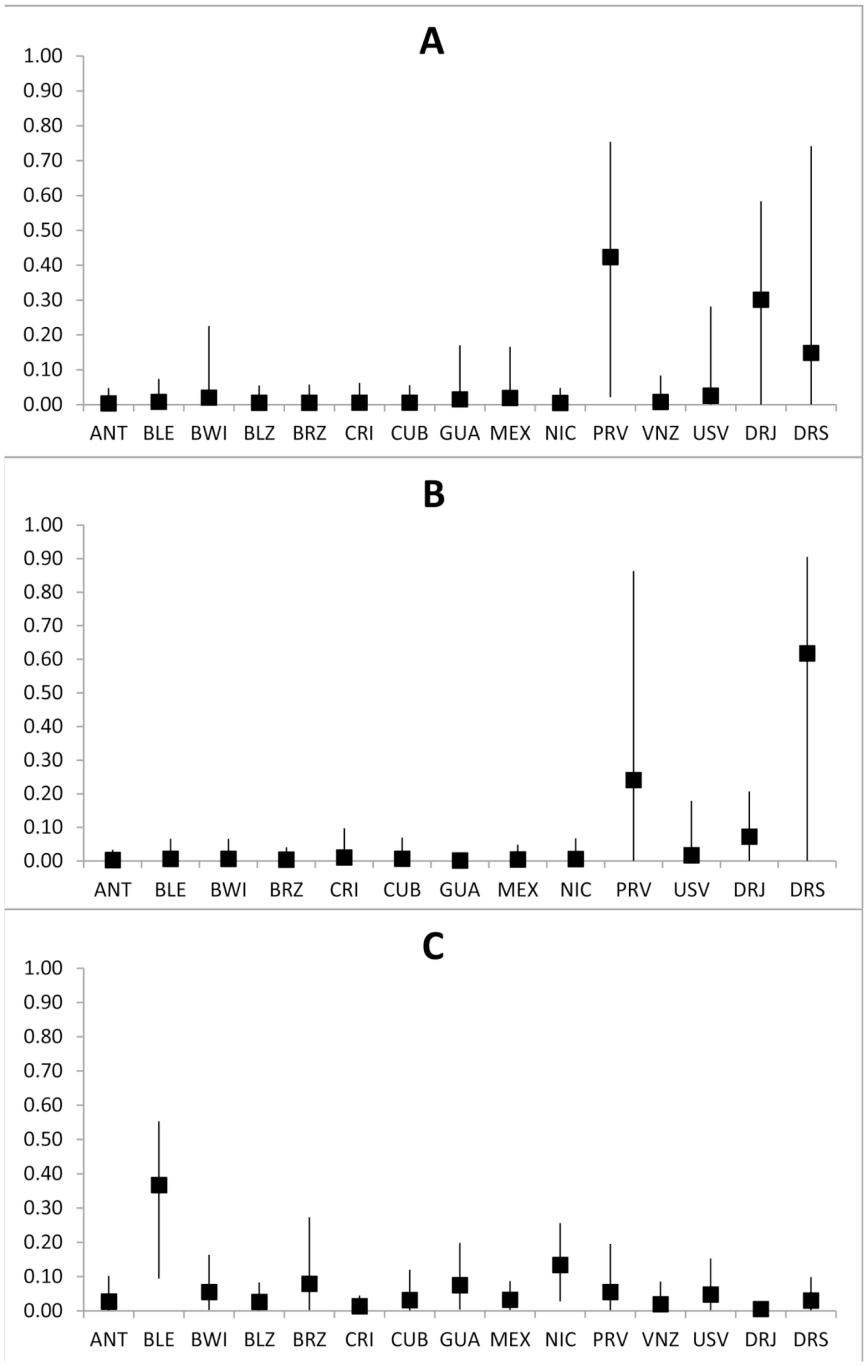
Mixed Stock analysis. Relative contribution and 95% confidence interval of each hawksbill turtle nesting area to the male aggregation of Mona Island (Puerto Rico) using A) the short (380_bp_) mtDNA fragment, B) the long (720_bp_) mtDNA fragment and C) to the SW Dominican Republic feeding ground using the short (380_bp_) mtDNA fragment. ANT: Antigua; BLE: Barbados Leeward; BWI: Barbados Windward; BLZ: Belize; BRZ: Brazil; CRI: Costa Rica; CUB: Cuba; GUA: Guadeloupe; MEX: Mexico; NIC: Nicaragua; PRV: Puerto Rico [35]; PRB: Puerto Rico [64]; USV: US Virgin Islands; VNZ: Venezuela; DRJ: Dominican Republic-Jaragua; DRS: Dominican Republic-Saona.

The *rookery centred* ‘many-to-many’ mixed stock analysis for Dominican Republic juveniles suggested that turtles originating from the two nesting areas are likely to have been distributed in foraging grounds across the Caribbean, although the 95% confidence intervals were very high (Table 4). The *mixed stock centred* analysis showed that the proportion of turtles in the genotyped Caribbean juvenile feeding grounds coming from Dominican Republic nesting populations is very low, being less than 0.01 from Jaragua National Park and less than 0.06 from Saona Island in all cases (Table 4). Despite these results, the mixed stock analysis with the short fragment showed that both Jaragua National Park (DRJ) and Saona Island (DRS) significantly contributed to the adult male aggregation in Puerto Rico, along with the breeding stocks from Puerto Rico itself, (DRJ: 0.30 (0.00–0.58), DRS: 0.15 (0.00–0.74), Figure 3A). However, the contribution of Saona Island exceeded that for Jaragua National Park when the new long fragment was considered (DRJ: 0.07 (0.00–0.21), DRS: 0.62 (0.00–0.91), Figure 3B). The population of Barbados leeward has the highest contribution of juveniles to the SW Dominican Republic feeding aggregation (Figure 3B).

**Table 4:** ‘Many to many’ mixed stock analysis results.

	*Rookery centred*		*Mixed stock centred*		
Mixed stock	DRJ	DRS	DRJ	DRS	*Reference*
1. Texas	6.9 (0.2–23.3)	2.9 (0.1–10.9)	0.8 (0.0–3.0)	2.2 (0.1–8.1)	[38]
2. Bahamas	7.5 (0.2–25.8)	5.1 (0.2–18.7)	0.6 (0.0–2.3)	2.6 (0.1–8.9)	[38]
3. Cuba D	8.8 (0.3–30.4)	9.4 (0.4–28.3)	0.7 (0.0–2.8)	5.2 (0.2–16.1)	[65]
4. Cuba B	7.6 (0.2–26.7)	8.2 (0.3–26.3)	0.4 (0.0–1.4)	2.6 (0.1–9.4)	[65]
5. Cuba A	7.6 (0.2–25.5)	6.3 (0.2–23.9)	0.3 (0.0–1.1)	1.5 (0.0–5.7)	[65]
6. Turk and Caicos	7.4 (0.2–23.1)	6.7 (0.2–22.5)	0.5 (0.0–1.9)	2.8 (0.1–9.4)	[42]
7. Cayman Islands	7.8 (0.2–26.7)	8.5 (0.2–27.8)	0.4 (0.0–1.7)	3.1 (0.1–10.0)	[41]
8. Dominican Republic	8.0 (0.3–27.0)	7.2 (0.2–23.0)	0.5 (0.0–2.0)	3.0 (0.1–9.8)	[38]
9. Puerto Rico residents	7.1 (0.2–25.2)	7.4 (0.2–26.5)	0.4 (0.0–1.7)	3.0 (0.1–11.2)	[35]
10. Puerto Rico recruits	7.8 (0.2–27.2)	11.6 (0.4–33.5)	0.3 (0.0–1.3)	3.5 (0.1–12.4)	[35]
11. Puerto Rico pooled	8.7 (0.2–27.8)	11.6 (0.4–31.9)	0.6 (0.0–2.1)	5.3 (0.2–15.3	[38], [65]
12. US Virgin Islands	7.4 (0.2–25.2)	7.0 (0.2–24.7)	0.3 (0.0–1.2)	2.1 (0.1–7.9)	[38]
13. Unknown	7.3 (0.2–26.0)	8.0 (0.3–28.2)	NA	NA	

*Rookery centred* analysis includes the percentage of Dominican Republic juveniles that use a mixed stock and an esteem of juveniles that disperse to *unknown* juvenile feeding grounds (last line). *Mixed stock centred* analysis includes the percentage of turtles from the mixed stock coming from Dominican Republic nesting populations. 95% confidence intervals are shown in brackets. NA: Not applicable.

### Lagrangian Buoys Dispersion

**Figure 4:**
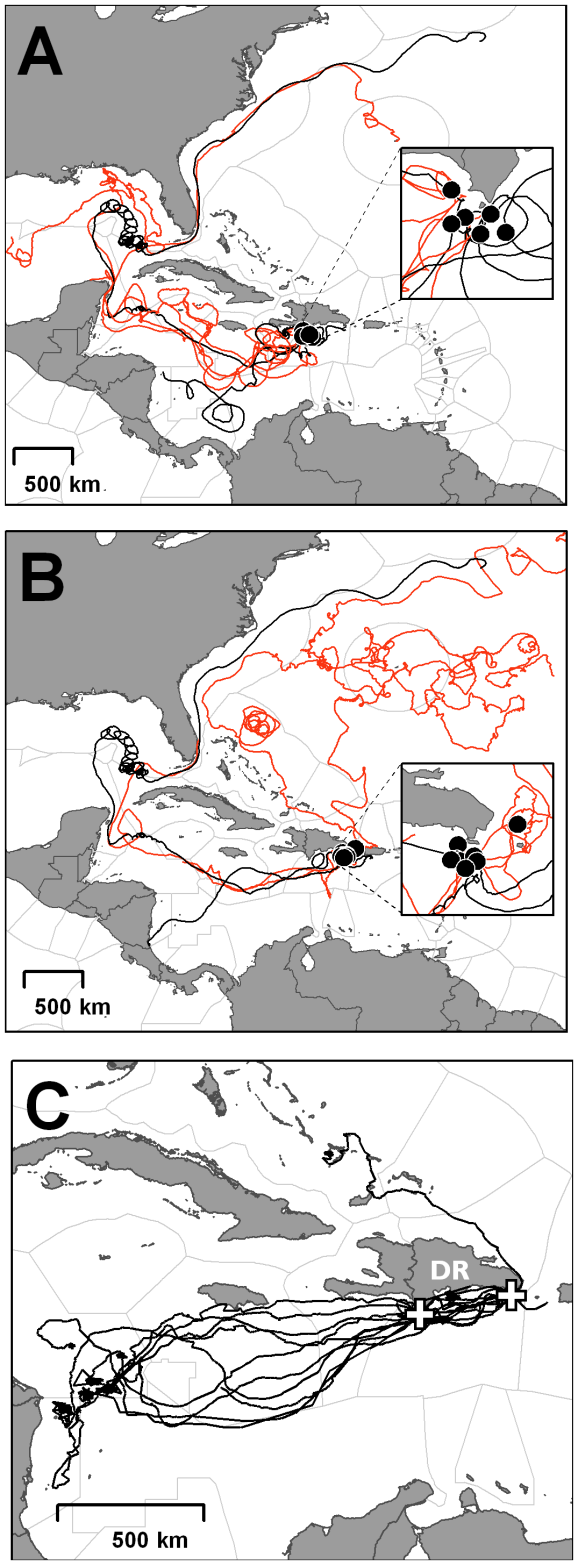
Tracks of passive drifter buoys and satellite traked turtles. (A, B) Tracks of passive drifter buoys on departure from the (A) Jaragua National Park or (B) Saona Island (starting point indicated with black circles). Red lines show tracks on departure during the nesting season. Figure (C) shows the migrations of ten satellite tracked adult female hawksbill turtles [102]. White crosses show deployment locations for satellite tracked turtles in Jaragua and Saona Island. Pale grey lines show the extent of the Exclusive Economic Zone for each Caribbean country. Note part (C) is to a different scale.

A total of 22 passive drifter buoys approached and/or left the Jaragua National Park and Saona Island areas (DRJ: n = 10; DRS: n = 11; both: n = 1) (Figure 4) between 1996 and 2010. All buoys that arrived at the Dominican Republic did so from the south-east of the island. They arrived year round, with 72.7% being recorded during the sampling period and with 45.5% arriving during the summer. However, buoys that left the Dominican Republic travelled in different directions: all those from Jaragua travelled south-west into the Caribbean (Figure 4A) while some of those from Saona Island (25%) travelled north into the open Atlantic (Figure 4B). These differences in the trajectories remained when only tracks during the nesting season were considered (Figure 4). Mean drifting time was 307 days but was highly variable between buoys (7–941 days). Mean drifting time of the buoys following departure from study area (Figure 4) was 265 days (10–883 days).

## Discussion

Conservation planning for mobile species such as marine turtles, depends on robust spatio-temporal information about the RMU of interest [13]. Since the MUs [29], which compose a RMU, may be sharing common reproductive or feeding areas (even if they are genetically isolated), the lack of information about many MUs may lead to poor or biased management effort. Such information gaps are particularly pertinent where threats vary among MUs [87]–[88].

### Green Turtle

Green turtles in the Dominican Republic [22] declined from *ca.* 260 green turtles nesting per year in the 1980s [14] to near extirpation at present [22], and precluded robust analysis of population structuring or connectivity. However, they appear to share a haplotype very common in Suriname and Aves Island, hence suggesting that population recovery through immigration could be possible in the future if these populations are still connected.

### Leatherback Turtle Population Structure

The leatherback turtle is thought to be the least philopatric of the marine turtles [89]–[91], and consequently genetic markers in the present study and others [61] have demonstrated high levels of population connectivity. The nesting populations of Awala-Yamalipo, Cayenne and Trinidad emerged as a distinct MU, separated from St Croix MU. The Dominican Republic nesting population from the present study was similar to the other Caribbean nesting populations forming part of a third MU in the region. The use of 711_bp_ sequences did not change the number of haplotypes in the Dominican Republic in contrast to previous studies, where some 496_bp_ haplotypes were subdivided into multiple 711_bp_ haplotypes [62]–[63]. Future work expanding the use of 711_bp_ sequences to other nesting and feeding areas is needed in order to test if longer sequences improve resolution of population structuring as they have for the loggerhead turtle [44].

The connection between the Dominican Republic leatherback population with several larger populations within the Atlantic would favour putative recovery through immigration [89]–[91]. However, unless ameliorated, the anthropogenic stressors that have contributed to population decline would likely affect immigrant turtles equally and hence our study site may act as a sink. The Costa Rica nesting aggregation, for example, is one of the largest populations in the Caribbean, but is thought to be declining despite some of the most heavily resourced conservation efforts in the world [90]. Sink areas like the Dominican Republic may have been partially responsible for such a decline and is worthy of future investigation. Finally, the genetic signature of important nesting sites in Colombia-Panama [92] still remains unknown, and hence their potential role as source populations within the Caribbean remains to be elucidated.

### Hawksbill Turtle Population Structure

Previous studies in the Caribbean have shown that the hawksbill turtle exhibits a high degree of philopatry resulting in fine scale population structure [38], [65]–[66], [93], sometimes at surprisingly small geographic scales [40]. The present study offers striking support for this, demonstrating that in the Dominican Republic, the two hawksbill populations were genetically distinct despite being separated by only 300 km straight-line distance. Hence, we strongly recommend finer scale sampling efforts in order to detect the genetic richness of a given territory as different MUs may be separated by much smaller distances [40] than classically thought [93].

Beyond the fine scale structuring detected within the Dominican Republic, the two populations are clearly isolated from almost all other Caribbean nesting populations with the exception of DRS with Puerto Rico, US Virgin Islands and Belize in terms of maternally inherited DNA (mtDNA). The nesting aggregation in Mona Island, Puerto Rico [35], [82] is only 68 km straight distance from Saona Island, in the Dominican Republic, while the US Virgin Islands are 410 km. The connection with Belize is more difficult to explain but this population is located at the same latitude as the Dominican Republic. A study of the loggerhead marine turtles in Florida [94] noted that haplotype frequencies were similar for nesting populations at similar magnetic fields, a putative homing cue for nesting females. However, the statistical significance between Belize and DRS disappeared only after FDR correction for one of the tests (P = 0.042 for the exact test) and the Belizean sample size was very small, and thus potentially misleading. Future analyses of the Belizean nesting population including more samples are needed to test if this connection is real or an artefact of sample size. The populations of Puerto Rico, US Virgin Islands and DRS exhibited some levels of connection with Barbados (windward), depending on the length of the marker and the statistical test used. This upper level of structuring was clearly detected by the PCA analysis and supports the AMOVA analyses conducted in previous studies [40]. In both cases, three clusters were clearly defined and the haplotype composition suggests an evolutionary origin for such structuring. The first group is characterized by the high frequency of the Q (380_bp_)/EiA23 (720_bp_) haplotype, the second by the high frequency of the F (380_bp_)/EiA11 (720_bp_) haplotype and the last by the high frequency of the A (380_bp_)/EiA1 (720_bp_) haplotype (Figure 2B, 2C). Based on data gathered using multiple techniques, the whole Caribbean has been proposed as a unique RMU for the hawksbill sea turtle [13], comprised of several different isolated MUs defined using genetic markers [35], [38], [40], [93]. Here we highlight the existence of an intermediate level of structuring associated with haplotype composition, as detected in previous studies [40]. Whether this intermediate level corresponds to a RMU or not needs to be tested in the future using other markers (such as microsatellites or SNPs) and combining the results with all available information of the species in the area.

The structuring of the Caribbean stocks has strong implications for the conservation of the hawksbill nesting populations of the Dominican Republic. The DRJ population is completely isolated, suggesting it is unlikely to receive females by immigration. The small population size [22] could favour the loss of the maternally inherited genetic diversity through mechanisms such as genetic drift or inbreeding. On the other hand, DRS population fate would appear to be linked to other nesting populations, especially Puerto Rico and US Virgin Islands (but also Barbados and perhaps Belize). However, this also means that the threats to the species detected in the area of Saona have potential impacts for these linked nesting aggregations. Fortunately, populations of hawksbill turtles nesting in Puerto Rico [82] and Barbados [82], [95] have increased recently. This increase may have a future positive effect on DRS nesting population through immigration of non-philopatric females from these two areas.

Regardless of female philopatry, hawksbill turtles appear to be highly mobile during the juvenile developmental phase. Blumenthal et al. [41] modelled the passive dispersal of virtual particles released in known hawksbill nesting areas during the hatchling dispersal phase and compared it with genetic markers to support the hypothesis that juvenile dispersion is highly dependent on current patterns. For the Greater Antilles ecoregion, the model predicted that the particles would divide in two well differentiated branches when arriving the Dominican Republic, one heading south-west and entering the Caribbean and the other heading north and north-west and entering the open Atlantic. Lagrangian particle modelling is an excellent approach to describe the general pattern of passive dispersal as it easily provides a statistically robust sample size, has a wide spatio-temporal coverage [49], [96] and it is possible to add behavioural components to the modelled particles [53], [97]–[98]. However, this approach is sensitive to model resolution [96] and sometimes fails in the detection of small scale current variations [49]. On the other hand, Lagrangian drifting buoys reflect high resolution drifting trajectories, but usually have a limited spatio-temporal coverage and sample sizes are much lower than modelling, so biases can be also found [49]. For these reasons it is desirable to contextualise findings with both techniques to study the effect of passive drifting in dispersal [49], [96]. The Lagrangian drifting buoys that departed from the Dominican Republic (present study) followed similar patterns than described in the Lagrangian particles model of Blumenthal et al. [41] but also showed that, depending on the release point, the buoys may take only the southern branch into the Caribbean (Jaragua National park, Figure 4A) or also enter the open Atlantic (Saona Island, Figure 4B). This subtle small scale variation on the current system may produce a differential dispersion of hatchlings from both Dominican Republic nesting populations, as hatchlings are known to be highly influenced by the dominant currents. However, marine turtle hatchlings and juveniles are also known to be able to contribute substantially to the net movement by weak directed swimming thus escaping from cold or highly predated areas [49], [53], [56], [97]–[98]. Thus, directed swimming of hatchlings from these areas could greatly affect the proportion of individuals entering the open Atlantic or the inner Caribbean sea. Recent papers proposed the ‘learned migration goal’ hypothesis (LMG) [51], [56] to explain how adult marine turtles can be influenced by the currents system despite being able to swim against them. Under the LMG theory, adult turtles would follow the preferred route from those learned as hatchlings and juveniles and fixed in a magnetic map [99]–[101]. Thus, the subtle oceanic differences detected within the vicinity of Dominican Republic nesting beaches would be reflected in females’ behaviour and could explain the significant genetic differences observed between Saona Island and Jaragua National Park nesting populations, irrespective of their proximity to one another. A recent telemetry study [102] showed that the nesting females that left the country (the majority from Saona Island) took the southwestern route after the nesting season, heading into the Caribbean towards the western Caribbean basin (Figure 4C). The only satellite tracked adult female to head northwards was deployed from Saona Island, supporting the idea that adult turtle migration may be still partially influenced by the oceanographic currents that affected them as hatchlings and juveniles [51], [56].

### Hawksbill Juvenile and Male Dispersion

Considering all these results and the putative dispersal mechanisms of the species [41], one may expect that DRJ juveniles would disperse mainly southwards towards inner Caribbean feeding grounds while DRS juveniles may disperse north and eastwards into the open Atlantic, Bahamas, Cuba (foraging ground D) or the Turks and Caicos Islands. Surprisingly, the *rookery centred* mixed stock analysis, did not support this hypothesis, as it predicted an homogeneous distribution of juveniles originating in both Dominican Republic nesting populations. Such mixed stock analysis usually yields wide confidence intervals in marine turtles due to the existence of common haplotypes and results may be taken with caution. Furthermore, the population sizes of the two Dominican Republic nesting populations [22] are one or two orders of magnitude lower than the most abundant nesting areas [82] and hence the production of hatchlings is likely much lower. As a consequence, the contribution of Dominican Republic populations to all the juvenile feeding grounds would be necessarily very low, as indicated by the *mixed stock centred* analysis. For instance, the juvenile feeding ground located near DRJ [38], [103] receives turtles mainly from Barbados, [40] in agreement with the particle dispersal model [41] and the drifter buoys that arrived in the area (*present study*), but it also receives turtles from Cuba. This means that any extreme mortality of this juvenile aggregation would have an impact on these populations.

The contribution of both Dominican Republic nesting populations to the Puerto Rico male aggregation is very clear. Satellite tracks of adult males and females in Puerto Rico showed that adults move from Puerto Rico and arrive to the vicinity of DRJ and DRS and remained there, possibly to breed [104]. The present study has shown that adult individuals from these three nesting aggregations share the same foraging areas. Given that DRJ is clearly a different genetic unit, the fact that adult males are using the same foraging areas may indicate the existence of common mating areas and opens the possibility of male mediated gene flow, as found for other sea turtle species [32]–[33], [105]. The use of biparentally inherited markers, such as microsatellites or SNPs, is needed to test the existence of male mediated gene flow in these areas.

### Genetic Variability of Leatherback and Hawksbill Nesting Populations

Historical measures of the genetic variability would be desirable in order to detect a recent loss of the genetic variability in the Dominican Republic nesting populations caused by the recent population decline [22]. Unfortunately, only recent measures from other populations can be obtained using current haplotype frequencies, and all populations have been affected to some extent by human activities. However, the conservation status and population sizes of marine turtle populations within the Caribbean are highly variable, so a comparison of the genetic variability among them would provide a relative measure of the genetic health of the studied populations. Thus, it is reasonable to conclude that the extreme recent reduction of the leatherback and hawksbill Dominican Republic nesting populations has not yet been reflected in a substantial loss of genetic variability, as they had values similar or higher to almost all other populations in the same area that have high population sizes or are increasing due to conservation efforts. The addition of measures of variability obtained from biparentally inherited markers would provide greater insight about how population reduction impacts in the genetic variability.

### Conclusions

One of the milestones in the conservation of endangered species is the detection and quantification of threats affecting declining populations. However, the contextualisation of these potential conservation sinks in a wider regional area is necessary when complex life histories and complex populations structures are present, such as in sea turtles [8]–[9]. The present study provides one such case study and goes beyond the detection of the threats of the Dominican Republic and the local decline [22]. The detection of fine scale structuring within Dominican Republic hawksbill populations, the isolated nature of the Jaragua National Park hawksbill nesting population, the establishment of migratory pathways involving the threatened Dominican Republic marine turtles and the use of common feeding grounds at different life stages has been crucial in understanding which populations might be affected by a local sink and which healthy populations might act as a source of individuals to help the recovery of threatened populations.

## Supporting Information

Table S1 **Haplotype frequencies of Caribbean Hawksbill marine turtle nesting populations.** Using the short (380 bp) fragment and including nesting population size (nests/year) used as a baseline for the Mixed Stock Analysis. Puerto Rico data from PRB [1] was not included in the baseline as PRV [2] included a larger sampling set from the same location. PS: Present Study.
(DOCX)

Table S2 **Haplotype frequencies of Caribbean Hawksbill marine turtle feeding grounds.** Using the short (380 bp) fragment.
(DOCX)

Table S3 **Haplotype frequencies of Caribbean Hawksbill marine turtle nesting populations.** Using the long (720 bp) fragment and including nesting population size (nests/year) used as a baseline for the Mixed Stock Analysis. PS: Present Study.
(DOCX)

## References

[1] Schmidt J (1923) Breeding places and migrations of the eel. Nature 111: 51–54.

[2] Campagna C, Piola AR, Marin MR, Lewis M, Fernandez T (2006) Southern elephant seal trajectories, fronts and eddies in the Brazil/Malvinas Confluence. Deep-Sea Res Part I-Oceanogr Res Pap 53: 1907–1924.

[3] Cotte C, Park YH, Guinet C, Bost CA (2007) Movements of foraging king penguins through marine mesoscale eddies. P Roy Soc B-Biol Sci 274: 2385–2391. 10.1098/rspb.2007.0775 PMC2274980 17669726

[4] Cotte C, d'Ovidio F, Chaigneau A, Levy M, Taupier-Letage I, et al (2011) Scale-dependent interactions of Mediterranean whales with marine dynamics. Limnol Oceanogr 56: 219–232.

[5] Horton TW, Holdaway RN, Zerbini AN, Hauser N, Garrigue C, et al (2011) Straight as an arrow: humpback whales swim constant course tracks during long-distance migration. Biol Lett 7: 674–679. 21508023 10.1098/rsbl.2011.0279 PMC3169072

[6] Sleeman JC, Meekan MG, Wilson SG, Polovina JJ, Stevens JD, et al (2010) To go or not to go with the flow: Environmental influences on whale shark movement patterns. J Exp Mar Biol Ecol 390: 84–98.

[7] Lohmann KJ, Witherington BE, Lohmann CMF, Salmon M (1997) Orientation, navigation and natal beach homing in sea turtles. In: Lutz PL, Musick JA, editors. The biology of sea turtles. Boca Raton: CRC Press. 107–136.

[8] Bowen BW, Karl SA (2007) Population genetics and phylogeography of sea turtles. Mol Ecol 16: 4886–4907. 17944856 10.1111/j.1365-294X.2007.03542.x

[9] Lee PLM (2008) Molecular ecology of marine turtles: New approaches and future directions. J Exp Mar Biol Ecol 356: 25–42.

[10] Hays GC, Scott R (2013) Global patterns for upper ceilings on migration distance in sea turtles and comparisons with fish, birds and mammals. Funct Ecol DOI: 10.1111/1365-2435.12073

[11] Hamann M, Godfrey MH, Seminoff JA, Arthur K, Barata PCR, et al (2010) Global research priorities for sea turtles: informing management and conservation in the 21st century. Endang Species Res 11: 245–269.

[12] DiMatteo AD, Fujioka E, Wallace BP, Hutchinson BJ, Cleary J, et al. (2009) SWOT Database Online. Available: http://seamap.env.duke.edu/swot.

[13] Wallace BP, DiMatteo AD, Hurley BJ, Finkbeiner EM, Bolten AB, et al (2010) Regional Management Units for Marine Turtles: A Novel Framework for Prioritizing Conservation and Research across Multiple Scales. Plos One 5 (12): e15465 doi:10.1371/journal.pone.0015465 10.1371/journal.pone.0015465 PMC3003737 21253007

[14] Ottenwalder JA (1981) Estudio preliminar sobre el estado, distribución, y biología reproductiva de las tortugas marinas en la República Dominicana. Tesis de Licenciatura, Universidad Autónoma de Santo domingo, Santo Domingo.

[15] Ross JP, Ottenwalder JA (1983) The leatherback sea turtle, *Dermochelys coriacea*, nesting in the Dominican Republic. Advances in herpetology and evolutionary biology Essays in honor of Ernest E Williams. 706–713.

[16] Dominguez TJ, Villalba AA (1994) Trade of hawksbill carapaces in Santo Domingo, Dominican Republic. In: Bjorndal A, Bolten A, Johnson DA. Eliazar PJ, editors. Proceedings of the fourteenth Annual Symposium on Sea turtle Biology and Conservation: 34–35.

[17] Mota JM, Leon YM (2003) Beliefs and perceptions associated with sea turtle products in the Dominican Republic. In: Pilcher NJ, editor. Proceedings of the twenty-third Annual Symposium on Sea Turtle Biology and Conservation: 197–199.

[18] Feliz P, Leon YM, Tomás J, Hierro K, Mateo A, et al.. (2008) Tortoiseshell trade in Santo Domingo, Dominican Republic: discouraging news for Caribbean hawksbill. In: Dean K, López Castro MC, editors. Proceedings of the 28th Annual sea turtle symposium, Loreto, Baja California Sur, Mexico: 247.

[19] Ottenwalder JA (1996) Conservation and Management of Sea Turtles in the Dominican Republic. CITES.

[20] Aucoin S, Leon YM (2007) Preliminary data on hawksbill turtle (*Eretmochelys imbricata*) bycatch in an artisanal gillnet used near Jaragua National Park, Dominican Republic. Proceedings of the Gulf and Caribbean Fisheries Institute 60: 169–172.

[21] Powell R, Inchaustegui SJ (2009) Conservation of the herpetofauna of the Dominican Republic. Appl Herpetol 6: 103–122.

[22] Revuelta O, León YM, Feliz P, Godley BJ, Raga A, et al (2012) Protected areas host important remnants of marine turtle nesting stocks in the Dominican Republic. Oryx 46: 348–358.

[23] Fleming EH (2001) Swimming against the Tide: recent surveys of exploitation, trade and management of marine turtles in the Northern Caribbean. Washington DC, USA: TRAFFIC North america.

[24] Stam S, Stam R (1992) Turtle trouble in the Dominican Republic. Marine Turtle Newsletter 57: 19.21.

[25] Reuter A, Allan C (2006) Tourists, turtles and trinkets: a look at the trade in marine turtle products in the Dominican Republic and Colombia. Washington, DC, USA: TRAFFIC North America.

[26] Leon YM (2004) Community impacts of coastal tourism in the Dominican Republic. PhD dissertation. Kingston, Rhode Island, USA: University of Rhode Island.

[27] Wielgus J, Cooper E, Torres R, Burke L (2010) Coastal capital: Dominican Republic. Case studies on the economic value of coastal ecosystems in the Dominican Republic. Washington DC, USA: World Resources Institute.

[28] Laurent L, Casale P, Bradai MN, Godley BJ, Gerosa G, et al (1998) Molecular resolution of marine turtle stock composition in fishery bycatch: a case study in the Mediterranean. Mol Ecol 7: 1529–1542. 9819906 10.1046/j.1365-294x.1998.00471.x

[29] Moritz C (1994) Defining Evolutionarily Significant Units for Conservation. Trends Ecol Evol 9: 373–375. 21236896 10.1016/0169-5347(94)90057-4

[30] Carreras C, Pont S, Maffucci F, Pascual M, Barcelo A, et al (2006) Genetic structuring of immature loggerhead sea turtles (*Caretta caretta*) in the Mediterranean Sea reflects water circulation patterns. Mar Biol 149: 1269–1279.

[31] Bowen BW, Abreu-Grobois A, Balazs GH, Kamezaki N, Limpus CJ, et al (1995) Trans-Pacific migrations of the loggerhead turtle (*Caretta caretta*) demonstrated with mitochondrial DNA markers. Proc Natl Acad Sci U S A 92: 3731–3734. 7731974 10.1073/pnas.92.9.3731 PMC42035

[32] Bowen BW, Bass AL, Soares L, Toonen RJ (2005) Conservation implications of complex population structure: lessons from the loggerhead turtle (*Caretta caretta*). Mol Ecol 14: 2389–2402. 15969722 10.1111/j.1365-294X.2005.02598.x

[33] FitzSimmons NN, Moritz C, Limpus CJ, Pope L, Prince R (1997) Geographic structure of mitochondrial and nuclear gene polymorphisms in Australian green turtle populations and male-biased gene flow. Genetics 147: 1843–1854. 9409840 10.1093/genetics/147.4.1843 PMC1208350

[34] Carreras C, Pascual M, Cardona L, Marco A, Bellido JJ, et al (2011) Living Together but Remaining Apart: Atlantic and Mediterranean Loggerhead Sea Turtles (*Caretta caretta*) in Shared Feeding Grounds. J Hered 102: 666–677. 21934114 10.1093/jhered/esr089

[35] Velez-Zuazo X, Ramos WD, van Dam RP, Diez CE, Abreu-Grobois A, et al (2008) Dispersal, recruitment and migratory behaviour in a hawksbill sea turtle aggregation. Mol Ecol 17: 839–853. 18208487 10.1111/j.1365-294X.2007.03635.x

[36] Bolten AB, Bjorndal KA, Martins HR, Dellinger T, Biscoito MJ, et al (1998) Transatlantic developmental migrations of loggerhead sea turtles demonstrated by mtDNA sequence analysis. Ecol Appl 8: 1–7.

[37] Bowen BW, Bass AL, Chow SM, Bostrom M, Bjorndal KA, et al (2004) Natal homing in juvenile loggerhead turtles (*Caretta caretta*). Mol Ecol 13: 3797–3808. 15548292 10.1111/j.1365-294X.2004.02356.x

[38] Bowen BW, Grant WS, Hillis-Starr Z, Shaver DJ, Bjorndal A, et al (2007) Mixed-stock analysis reveals the migrations of juvenile hawksbill turtles (*Eretmochelys imbricata*) in the Caribbean Sea. Mol Ecol 16: 49–60. 17181720 10.1111/j.1365-294X.2006.03096.x

[39] Monzon-Arguello C, Rico C, Carreras C, Calabuig P, Marco A, et al (2009) Variation in spatial distribution of juvenile loggerhead turtles in the eastern Atlantic and western Mediterranean Sea. J Exp Mar Biol Ecol 373: 79–86.

[40] Browne D, Horrocks J, Abreu-Grobois A (2010) Population subdivision in hawksbill turtles nesting on Barbados, west Indies, determined from mitochondrial DNA control region sequences. Conserv Genet 11: 1541–1546.

[41] Blumenthal JM, Abreu-Grobois FA, Austin TJ, Broderick AC, Bruford MW, et al (2009) Turtle groups or turtle soup: dispersal patterns of hawksbill turtles in the Caribbean. Molecular Ecology 18: 4841–4853. 19889039 10.1111/j.1365-294X.2009.04403.x

[42] Richardson PB, Bruford MW, Calosso MC, Campbell LM, Clerveaux W, et al (2009) Marine Turtles in the Turks and Caicos Islands: Remnant Rookeries, Regionally Significant Foraging Stocks, and a Major Turtle Fishery. Chelonian Conserv Bi 8: 192–207.

[43] Bolker BM, Okuyama T, Bjorndal KA, Bolten AB (2007) Incorporating multiple mixed stocks in mixed stock analysis: 'many-to-many' analyses. Mol Ecol 16: 685–695. 17284204 10.1111/j.1365-294X.2006.03161.x

[44] Monzon-Arguello C, Rico C, Naro-Maciel E, Varo-Cruz N, Lopez P, et al (2010) Population structure and conservation implications for the loggerhead sea turtle of the Cape Verde Islands. Conserv Genet 11: 1871–1884.

[45] Monzon-Arguello C, Lopez-Jurado LF, Rico C, Marco A, Lopez P, et al (2010) Evidence from genetic and Lagrangian drifter data for transatlantic transport of small juvenile green turtles. J Biogeogr 37: 1752–1766.

[46] Bass AL, Epperly SP, Braun-McNeill J (2006) Green turtle (*Chelonia mydas*) foraging and nesting aggregations in the Caribbean and Atlantic: Impact of currents and behavior on dispersal. J Hered 97: 346–354. 16782781 10.1093/jhered/esl004

[47] Luke K, Horrocks JA, LeRoux RA, Dutton PH (2004) Origins of green turtle (*Chelonia mydas*) feeding aggregations around Barbados, West Indies. Mar Biol 144: 799–805.

[48] Okuyama T, Bolker BM (2005) Combining genetic and ecological data to estimate sea turtle origins. Ecol Appl 15: 315–325.

[49] Fossette S, Putman NF, Lohmann KJ, Marsh R, Hays GC (2012) A biologist’s guide to assessing ocean currents: a review. Mar Ecol Prog Ser 457: 285–301.

[50] Hays GC, Marsh R (1997) Estimating the age of juvenile loggerhead sea turtles in the North Atlantic. Can J Zool-Rev Can Zool 75: 40–46.

[51] Hays GC, Fossette S, Katselidis KA, Mariani P, Schofield G (2010) Ontogenetic development of migration: Lagrangian drift trajectories suggest a new paradigm for sea turtles. J R Soc Interface 7: 1319–1327. 20236958 10.1098/rsif.2010.0009 PMC2894886

[52] Botsford LW, White JW, Coffroth MA, Paris CB, Planes S, et al (2009) Connectivity and resilience of coral reef metapopulations in marine protected areas: matching empirical efforts to predictive needs. Coral Reefs 28: 303–305. 10.1007/s00338-009-0466-z PMC3402229 22833699

[53] Scott R, Marsh R, Hays GC (2012) A little movement orientated to the geomagnetic field makes a big difference in strong flows. Mar Biol 159: 481–488.

[54] Shillinger GL, Di Lorenzo E, Luo H, Bograd SJ, Hazen EL, et al (2012) On the dispersal of leatherback turtle hatchlings from Mesoamerican nesting beaches. P Roy Soc B-Biol Sci 279: 2391–2395. 10.1098/rspb.2011.2348 PMC3350667 22378803

[55] Shillinger GL, Hazen EL, Bailey H, Bograd SJ, Godley B, et al (2012) Tagging through the stages: ontogeny in biologging. Mar Ecol Prog Ser 457: 163–301.

[56] Gaspar P, Benson SR, Dutton PH, Reveillere A, Jacob G, et al (2012) Oceanic dispersal of juvenile leatherback turtles: going beyond passive drift modeling. Mar Ecol Prog Ser 457: 265–284.

[57] Godley BJ, Barbosa C, Bruford M, Broderick AC, Catry P, et al (2010) Unravelling migratory connectivity in marine turtles using multiple methods. J Appl Ecol 47: 769–778.

[58] Garofalo L, Mingozzi T, Mico A, Novelletto A (2009) Loggerhead turtle (*Caretta caretta*) matrilines in the Mediterranean: further evidence of genetic diversity and connectivity. Mar Biol 156: 2085–2095.

[59] Abreu-Grobois A, Horrocks J, Formia A, Leroux R, Velez-Zuazo X, et al.. (2006) New mtDNA dloop primers which work for a variety of marine turtle species may increase the resolution capacity of mixed stock analysis. In: Frick MG, Panagopoulou A, Rees A, Williams KL, editors; Proceedings of the twenty-six Annual Symposium on Sea Turtle Biology and Conservation, Crete, Greece: 179.

[60] Hall TA (1999) BioEdit: a user-friendly biological sequence alignment editor and analysis program for Windows 95/98/NT. Nucleic Acids Symposium Series 41: 95–98.

[61] Dutton PH, Bowen BW, Owens DW, Barragan A, Davis SK (1999) Global phylogeography of the leatherback turtle (*Dermochelys coriacea*). J Zool 248: 397–409.

[62] Vargas SM, Araujo FCF, Monteiro DS, Estima SC, Almeida AP, et al (2008) Genetic diversity and origin of leatherback turtles (*Dermochelys coriacea*) from the Brazilian coast. J Hered 99: 215–220. 18252731 10.1093/jhered/esm120

[63] Molfetti E, Vilaça ST, Georges J-Y, Plot V, Delcroix E, et al (2013) Recent Demographic History and Present Fine-Scale Structure in the Northwest Atlantic Leatherback (*Dermochelys coriacea*) Turtle Population. Plos One 8 (3): e58061 doi:58010.51371/journal.pone.0058061 10.1371/journal.pone.0058061 PMC3596356 23516429

[64] Bass AL, Good DA, Bjorndal KA, Richardson JI, Hillis ZM, et al (1996) Testing models of female reproductive migratory behaviour and population structure in the Caribbean hawksbill turtle, *Eretmochelys imbricata*, with mtDNA sequences. Mol Ecol 5: 321–328. 8688954

[65] Diaz-Fernandez R, Okayama T, Uchiyama T, Carrillo E, Espinosa G, et al (1999) Genetic sourcing for the hawksbill turtle, *Eretmochelys imbricata*, in the northern Caribbean region. Chelonian Conserv Bi 3: 296–300.

[66] Troeng S, Dutton PH, Evans D (2005) Migration of hawksbill turtles *Eretmochelys imbricata* from Tortuguero, Costa Rica. Ecography 28: 394–402.

[67] Leroux RA, Dutton PH, Abreu-Grobois FA, Lagueux CJ, Campbell CL, et al (2012) Re-examination of Population Structure and Phylogeography of Hawksbill Turtles in the Wider Caribbean Using Longer mtDNA Sequences. J Hered 103: 806–820. 23045611 10.1093/jhered/ess055

[68] Rolf DA, Bentzen P (1989) The statistical analysis of mitochondrial DNA polymorphisms: X^2^ and the problem of small samples. Mol Biol Evol 6: 539–545. 2677600 10.1093/oxfordjournals.molbev.a040568

[69] Zaykin DV, Pudovkin AI (1993) Two programs to estimate the significance of X^2^ values using pseudoprobability tests. J Hered 84: 152.

[70] Goudet J, Raymond M, de Meeüs T (1996) Testing differentiation in diploid populations. Genetics 144: 1931–1938. 10.1093/genetics/144.4.1933 PMC1207740 8978076

[71] Nei M (1982) Evolution of Human Races at the Gene Level. In: Bonne-Tamir B, Cohen T, Goodman RM, editors. Human Genetics Part A: The Unfolding Genome;. New York, NY: Alan R. Liss. 167–181. 7163193

[72] Excoffier L, Laval G, Schneider S (2005) Arlequin (version 3.0): An integrated software package for population genetics data analysis. Evol Bioinform 1: 47–50. PMC2658868 19325852

[73] Fu YX (1997) Statistical tests of neutrality of mutations against population growth, hitchhiking and background selection. Genetics 147: 915–925. 9335623 10.1093/genetics/147.2.915 PMC1208208

[74] Rozas J, Sanchez-DelBarrio JC, Messeguer X, Rozas R (2003) DnaSP, DNA polymorphism analyses by the coalescent and other methods. Bioinf 19: 2496–2497. 10.1093/bioinformatics/btg359 14668244

[75] Peakall R, Smouse PE (2006) GENALEX 6: genetic analysis in Excel. Population genetic software for teaching and research. Mol Ecol Notes 6: 288–295. 10.1093/bioinformatics/bts460 PMC3463245 22820204

[76] Moran MD (2003) Arguments for rejecting the sequential Bonferroni in ecological studies. Oikos 100: 403–405.

[77] Cabin RJ, Mitchell RJ (2000) To Bonferroni or not to bonferroni: when and how are the questions. Bull Ecol Soc Am 81: 246–248.

[78] Narum SR (2006) Beyond Bonferroni: Less conservative analyses for conservation genetics. Conserv Genet 7: 783–787.

[79] Monzon-Arguello C, Loureiro NS, Delgado C, Marco A, Lopes JM, et al (2011) Principe island hawksbills: Genetic isolation of an eastern Atlantic stock. J Exp Mar Biol Ecol 407: 345–354.

[80] Monzon-Arguello C, Rico C, Marco A, Lopez P, Felipe Lopez-Jurado L (2010) Genetic characterization of eastern Atlantic hawksbill turtles at a foraging group indicates major undiscovered nesting populations in the region. J Exp Mar Biol Ecol 387: 9–14.

[81] Bass AL, Epperly SP, Braun-McNeill J (2004) Multi-year analysis of stock composition of a loggerhead turtle (*Caretta caretta*) foraging habitat using maximum likelihood and Bayesian methods. Conserv Genet 5: 783–796.

[82] Mortimer JA, Donnelly M (2007) Marine Turtle Specialist Group 2007 IUCN Red List status assessment Hawksbill turtle (*Eretmochelys imbricata*). Available: http://www.iucn-mtsg.org/red_list/ei/index.shtml.

[83] Encalada SE, Lahanas PN, Bjorndal KA, Bolten AB, Miyamoto MM, et al (1996) Phylogeography and population structure of the Atlantic and Mediterranean green turtle *Chelonia mydas*: A mitochondrial DNA control region sequence assessment. Mol Ecol 5: 473–483. 8794558

[84] Lahanas PN, Bjorndal KA, Bolten AB, Encalada SE, Miyamoto MM, et al (1998) Genetic composition of a green turtle (*Chelonia mydas*) feeding ground population: evidence for multiple origins. Mar Biol 130: 345–352.

[85] Bjorndal KA, Bolten AB, Troeng S (2005) Population structure and genetic diversity in green turtles nesting at Tortuguero, Costa Rica, based on mitochondrial DNA control region sequences. Mar Biol 147: 1449–1457.

[86] Formia A, Godley BJ, Dontaine JF, Bruford MW (2006) Mitochondrial DNA diversity and phylogeography of endangered green turtle (*Chelonia mydas*) populations in Africa. Conserv Genet 7: 353–369.

[87] Pulliam HR (1988) Sources, sinks, and population regulation. Am Nat 132: 652–661.

[88] Pulliam HR, Danielson BJ (1991) Sources, sinks, and habitat selection - a landscape perspective on Population-Dynamics. Am Nat 137: S50–S66.

[89] Ordonez C, Troeng S, Meylan A, Meylan P, Ruiz A (2007) Chiriqui Beach, Panama, the most important leatherback nesting beach in Central America. Chelonian Conserv Bi 6: 122–126.

[90] Troeng S, Harrison E, Evans D, de Haro A, Vargas E (2007) Leatherback turtle nesting trends and threats at Tortuguero, Costa Rica. Chelonian Conserv Bi 6: 117–122.

[91] Chacon-Chaverri D, Eckert KL (2007) Leatherback sea turtle nesting at Gandoca Beach in Caribbean Costa Rica: Management recommendations from fifteen years of conservation. Chelonian Conserv Bi 6: 101–110.

[92] Patino-Martinez J, Marco A, Quinones L, Godley B (2008) Globally significant nesting of the leatherback turtle (*Dermochelys coriacea*) on the Caribbean coast of Colombia and Panama. Biol Conserv 141: 1982–1988.

[93] Bass AL (1999) Genetic analysis to elucidate the natural history and behavior of hawksbill turtles (*Eretmochelys imbricata*) in the wider Caribbean: A review and re-analysis. Chelonian Conserv Bi 3: 195–199.

[94] Shamblin BM, Dodd MG, Bagley DA, Ehrhart LM, Tucker AD, et al (2011) Genetic structure of the southeastern United States loggerhead turtle nesting aggregation: evidence of additional structure within the peninsular Florida recovery unit. Mar Biol 158: 571–587.

[95] Beggs J, Horrocks J, Krueger B (2007) Increase in hawksbill sea turtle *Eremochelys imbricata* nesting in Barbados, West Indies. Endang Species Res 3: 159–168.

[96] Putman NF, He R (2013) Tracking the long-distance dispersal of marine organisms: sensitivity to ocean model resolution. J R Soc Interface 10: 20120979. 23349437 10.1098/rsif.2012.0979 PMC3627105

[97] Putman NF, Verley P, Shay TJ, Lohmann KJ (2012) Simulating transoceanic migrations of young loggerhead sea turtles: merging magnetic navigation behavior with an ocean circulation model. J Exp Biol 215: 1863–1870. 22573765 10.1242/jeb.067587

[98] Putman NF, Scott R, Verley P, Marsh R, Hays GC (2012) Natal site and offshore swimming influence fitness and long-distance ocean transport in young sea turtles. Mar Biol 159: 2117–2126.

[99] Lohmann KJ, Hester JT, Lohmann CMF (1999) Long-distance navigation in sea turtles. Ethol Ecol Evol 11: 1–23.

[100] Lohmann KJ, Putman NF, Lohmann CMF (2008) Geomagnetic imprinting: A unifying hypothesis of long-distance natal homing in salmon and sea turtles. Proc Natl Acad Sci U S A 105: 19096–19101. 19060188 10.1073/pnas.0801859105 PMC2614721

[101] Freake MJ, Muheim R, Phillips JB (2006) Magnetic maps in animals: A theory comes of age? Q Rev Biol 81: 327–347. 17240727 10.1086/511528

[102] Hawkes LA, Tomás J, Revuelta O, Leon YM, Blumenthal JM, et al (2012) Migratory patterns in hawksbill turtles described by satellite tracking. Mar Ecol Prog Ser 461: 223–232.

[103] Leon YM, Diez CE (1999) Population structure of hawksbill turtles on a foraging ground in the Dominican Republic. Chelonian Conserv Bi 3: 230–236.

[104] Van Dam RP, Diez CE, Balazs GH, Colon LAC, McMillan WO, et al (2008) Sex-specific migration patterns of hawksbill turtles breeding at Mona Island, Puerto Rico. Endang Species Res 4: 85–94.

[105] Carreras C, Pascual M, Cardona L, Aguilar A, Margaritoulis D, et al (2007) The genetic structure of the loggerhead sea turtle (*Caretta caretta*) in the Mediterranean as revealed by nuclear and mitochondrial DNA and its conservation implications. Conserv Genet 8: 761–775.

[106] SEATURTLE.ORG Maptool (2002) Available: http://www.seaturtle.org/maptool/.SEATURTLE.ORG, Inc. Accessed 2013 Mar 18.

[107] Meylan AB, Donnelly M (1999) Status justification for listing the hawksbill turtle (*Eretmochelys imbricata*) as Critically Endangered on the 1996 IUCN Red List of Threatened Animals. Chelonian Conserv Bi 3: 200–224.

