# Correction: Contextualising the Last Survivors: Population Structure of Marine Turtles in the Dominican Republic

**DOI:** 10.1371/annotation/e64dde75-a0bd-48ac-aa9a-be1fedf24898

**Published:** 2014-01-06

**Authors:** Carlos Carreras, Brendan J. Godley, Yolanda M. León, Lucy A. Hawkes, Ohiana Revuelta, Juan A. Raga, Jesús Tomás

Errors were introduced in the preparation of this article for publication. The correct totals for DRJ and DRS in Table 1 are 15 and 33, respectively. 

