# Supplementary material for: Contextualising the Last Survivors: Population Structure of Marine Turtles in the Dominican Republic
Source: PLoS One. 2013 Jun 19;8(6):e66037. doi: 10.1371/journal.pone.0066037 (PMC3686877; doi:10.1371/journal.pone.0066037)
Supplement: Table S1 — Haplotype frequencies of Caribbean Hawksbill marine turtle nesting populations. Using the short (380 bp) fragment and including nesting population size (nests/year) used as a baseline for the Mixed Stock Analysis. Puerto Rico data from PRB [1] was not included in the baseline as PRV [2] included a larger sampling set from the same location. PS: Present Study. (DOCX) [file pone.0066037.s001.docx]

|  | **Reference** | **A** | **B** | **F** | **G** | **H** | **I** | **L** | **N** | **O** | **P** | **Q** | **alpha** | **gamma** | **Cu3** | **Cu4** | **f** | **EiA65** | **EiA62** | **TOTAL** | **Nests/year** | **Reference** |
| --- | --- | --- | --- | --- | --- | --- | --- | --- | --- | --- | --- | --- | --- | --- | --- | --- | --- | --- | --- | --- | --- | --- |
| **Atigua (ANT)** | [3] | 42 | 29 | 1 |  |  |  |  |  |  |  |  |  |  |  |  |  |  |  | 72 | 203 | [3] |
| **Barbados L. (BLE)** | [4] | 54 |  |  |  |  |  |  |  |  |  |  |  |  |  |  |  |  |  | 54 | 1504 | [3] |
| **Barbados W. (BWI)** | [4] | 3 |  | 27 |  |  |  |  |  |  |  |  |  |  |  |  |  |  |  | 30 | 150 | [3] |
| **Belize (BLZ)** | [5] |  |  | 11 | 1 | 1 | 1 |  |  |  |  |  |  |  |  |  |  |  |  | 14 | 50 | [6] |
| **Brazil (BRZ)** | [3] | 56 |  |  |  |  |  |  |  |  |  |  |  |  |  |  | 9 |  | 1 | 66 | 304 | [3] |
| **Costa Rica (CRI)** | [3] |  |  | 36 | 5 |  |  | 6 |  |  |  |  | 12 |  |  | 1 |  |  |  | 60 | 25 | [3] |
| **Cuba (CUB)** | [3] | 62 |  | 1 |  |  |  |  |  |  |  |  |  | 5 | 1 | 1 |  |  |  | 70 | 130 | [3] |
| **Guadeloupe (GUA)** | [3] | 2 |  | 71 | 1 |  |  |  |  |  |  |  |  |  |  |  |  |  |  | 74 | 151 | [3] |
| **Mexico (MEX)** | [2] |  |  |  |  |  |  |  |  |  | 1 | 52 |  |  |  |  |  |  |  | 53 | 311 | [3] |
| **Nicaragua (NIC)** | [3] |  |  | 59 |  |  |  |  |  |  |  | 16 | 19 |  |  |  |  | 1 |  | 95 | 205 | [3] |
| **Puerto Rico (PRV)** | [2] | 3 |  | 62 |  |  |  | 1 | 34 | 6 |  | 3 |  |  |  |  |  |  |  | 109 | 740 | [3] |
| **Venezuela (VNZ)** | [7] | 7 |  |  |  |  |  |  |  |  |  |  |  |  |  |  |  |  |  | 7 | 159 | [6] |
| **US Virgin Isl. (USV)** | [3] | 8 | 2 | 52 |  |  |  |  | 4 |  |  | 1 |  |  |  |  |  |  |  | 67 | 158 | [3] |
| **D.R. Jaragua (DRJ)** | PS | 1 |  | 5 |  |  |  | 1 |  |  |  | 8 |  |  |  |  |  |  |  | 15 | 14.6 | [8] |
| **D.R. Saona (DRS)** | PS | 3 |  | 22 |  |  |  | 2 | 6 |  |  |  |  |  |  |  |  |  |  | 33 | 100 | [8] |
| **TOTAL** |  | 241 | 31 | 347 | 7 | 1 | 1 | 10 | 44 | 6 | 1 | 80 | 31 | 5 | 1 | 2 | 9 | 1 | 1 | 819 |  |  |

References

1. Bass AL (1999) Genetic analysis to elucidate the natural history and behavior of hawksbill turtles (*Eretmochelys imbricata*) in the wider Caribbean: A review and re-analysis. Chelonian Conserv Bi 3: 195-199.

2. Velez-Zuazo X, Ramos WD, van Dam RP, Diez CE, Abreu-Grobois A, et al. (2008) Dispersal, recruitment and migratory behaviour in a hawksbill sea turtle aggregation. Mol Ecol 17: 839-853.

3. Leroux RA, Dutton PH, Abreu-Grobois FA, Lagueux CJ, Campbell CL, et al. (2012) Re-examination of Population Structure and Phylogeography of Hawksbill Turtles in the Wider Caribbean Using Longer mtDNA Sequences. J Hered 103: 806-820.

4. Browne D, Horrocks J, Abreu-Grobois A (2010) Population subdivision in hawksbill turtles nesting on Barbados, west Indies, determined from mitochondrial DNA control region sequences. Conserv Genet 11: 1541-1546.

5. Meylan AB, Donnelly M (1999) Status justification for listing the hawksbill turtle (*Eretmochelys imbricata*) as Critically Endangered on the 1996 IUCN Red List of Threatened Animals. Chelonian Conserv Bi 3: 200-224.

6. Mortimer JA, Donnelly M (2007) Marine Turtle Specialist Group 2007 IUCN Red List status assessment Hawksbill turtle (*Eretmochelys imbricata*). Available at <http://www.iucn-mtsg.org/red_list/ei/index.shtml>.

7. Bowen BW, Grant WS, Hillis-Starr Z, Shaver DJ, Bjorndal A, et al. (2007) Mixed-stock analysis reveals the migrations of juvenile hawksbill turtles (*Eretmochelys imbricata*) in the Caribbean Sea. Mol Ecol 16: 49-60.

8. Revuelta O, León YM, Feliz P, Godley BJ, Raga A, et al. (2012) Protected areas host important remnants of marine turtle nesting stocks in the Dominican Republic. Oryx 46: 348-358.
