# Supplementary material for: Contextualising the Last Survivors: Population Structure of Marine Turtles in the Dominican Republic
Source: PLoS One. 2013 Jun 19;8(6):e66037. doi: 10.1371/journal.pone.0066037 (PMC3686877; doi:10.1371/journal.pone.0066037)
Supplement: Table S2 — Haplotype frequencies of Caribbean Hawksbill marine turtle feeding grounds. Using the short (380 bp) fragment. (DOCX) [file pone.0066037.s002.docx]

|  | **Reference** | **A** | **B** | **F** | **G** | **J** | **L** | **N** | **P** | **Q** | **alpha** | **Beta** | **gamma** | **CU3** | **CU4** | **Cum** | **b** | **n** | **DR1** | **DR2** | **EATL** | **Ei-A53** | **Ei-A72** | **TOTAL** |
| --- | --- | --- | --- | --- | --- | --- | --- | --- | --- | --- | --- | --- | --- | --- | --- | --- | --- | --- | --- | --- | --- | --- | --- | --- |
| **1.Texas (TEX)** | [1] |  |  | 3 |  |  |  |  |  | 39 |  |  |  |  |  |  |  |  |  |  |  |  |  | 42 |
| **2.Bahamas (BAH)** | [1] | 28 | 1 | 20 |  | 2 |  |  | 1 | 21 | 1 |  |  | 3 |  |  |  |  | 1 |  |  |  |  | 78 |
| **3.Cuba A (CUA)** | [2] | 28 |  | 6 |  |  |  |  |  | 3 | 2 |  | 1 | 2 |  |  |  |  | 1 |  |  |  |  | 43 |
| **4.Cuba B (CUB)** | [2] | 46 |  | 34 |  |  | 1 | 10 |  | 11 | 1 |  | 5 | 2 |  |  |  |  | 1 |  |  |  |  | 111 |
| **5.Cuba D (CUD)** | [2] | 18 | 1 | 13 | 1 |  | 2 | 3 |  | 8 | 2 |  | 5 | 2 | 1 |  |  |  |  |  |  |  |  | 56 |
| **6.Turk and Caicos (TCI)** | [3] | 12 | 1 | 12 |  |  |  | 1 |  | 9 | 1 |  |  |  |  |  | 1 |  |  |  |  | 1 |  | 38 |
| **7.Cayman Islands (CAY)** | [4] | 44 | 1 | 25 |  |  | 1 | 1 |  | 11 | 2 |  |  | 1 |  |  | 4 |  |  |  |  |  | 2 | 92 |
| **8.Dominican Republic (DRE)** | [1] | 42 |  | 30 | 2 | 1 | 1 |  |  | 6 | 6 |  |  |  |  |  |  |  | 1 | 1 |  |  |  | 90 |
| **9.Puerto Rico recruits (REC)** | [5] | 31 | 1 | 23 |  |  | 2 | 1 |  | 2 | 2 |  |  |  |  |  |  |  |  |  |  |  |  | 62 |
| **10.Puerto Rico residents (RES)** | [5] | 19 | 1 | 25 |  |  |  | 2 | 1 | 4 | 1 |  |  | 2 |  |  |  | 1 |  |  |  |  |  | 56 |
| **11.Puerto Rico pooled (PRP)** | [1-2] | 38 | 2 | 62 |  |  | 3 | 8 |  | 18 | 5 | 1 | 1 |  |  |  |  |  |  |  |  |  |  | 138 |
| **12.US Virgin Islands (USV)** | [1] | 28 | 2 | 17 |  |  |  | 6 |  | 9 | 3 |  | 1 |  |  | 1 |  |  |  | 1 | 1 |  |  | 69 |
| **TOTAL** |  | 334 | 10 | 270 | 3 | 3 | 10 | 32 | 2 | 141 | 26 | 1 | 13 | 12 | 1 | 1 | 5 | 1 | 4 | 2 | 1 | 1 | 2 | 875 |

References

1. Bowen BW, Grant WS, Hillis-Starr Z, Shaver DJ, Bjorndal A, et al. (2007) Mixed-stock analysis reveals the migrations of juvenile hawksbill turtles (*Eretmochelys imbricata*) in the Caribbean Sea. Mol Ecol 16: 49-60.

2. Diaz-Fernandez R, Okayama T, Uchiyama T, Carrillo E, Espinosa G, et al. (1999) Genetic sourcing for the hawksbill turtle, *Eretmochelys imbricata*, in the northern Caribbean region. Chelonian Conserv Bi 3: 296-300.

3. Richardson PB, Bruford MW, Calosso MC, Campbell LM, Clerveaux W, et al. (2009) Marine Turtles in the Turks and Caicos Islands: Remnant Rookeries, Regionally Significant Foraging Stocks, and a Major Turtle Fishery. Chelonian Conserv Bi 8: 192-207.

4. Blumenthal JM, Abreu-Grobois FA, Austin TJ, Broderick AC, Bruford MW, et al. (2009) Turtle groups or turtle soup: dispersal patterns of hawksbill turtles in the Caribbean. Mol Ecol 18: 4841-4853.

5. Velez-Zuazo X, Ramos WD, van Dam RP, Diez CE, Abreu-Grobois A, et al. (2008) Dispersal, recruitment and migratory behaviour in a hawksbill sea turtle aggregation. Mol Ecol 17: 839-853.
