# Supplementary material for: Contextualising the Last Survivors: Population Structure of Marine Turtles in the Dominican Republic
Source: PLoS One. 2013 Jun 19;8(6):e66037. doi: 10.1371/journal.pone.0066037 (PMC3686877; doi:10.1371/journal.pone.0066037)
Supplement: Table S3 — Haplotype frequencies of Caribbean Hawksbill marine turtle nesting populations. Using the long (720 bp) fragment and including nesting population size (nests/year) used as a baseline for the Mixed Stock Analysis. PS: Present Study. (DOCX) [file pone.0066037.s003.docx]

|  | **Reference** | **EiA1** | **EiA61** | **EiA2** | **EiA52** | **EiA3** | **EiA9** | **EiA11** | **EiA84** | **EiA12** | **EiA13** | **EiA18** | **EiA47** | **EiA20** | **EiA21** | **EiA22** | **EiA23** | **EiA41** | **EiA43** | **EiA29** | **EiA30** | **EiA32** | **EiA62** | **EiA65** | **TOTAL** | **Nests/year** | **Reference** |
| --- | --- | --- | --- | --- | --- | --- | --- | --- | --- | --- | --- | --- | --- | --- | --- | --- | --- | --- | --- | --- | --- | --- | --- | --- | --- | --- | --- |
| **Atigua (ANT)** | [1] | 42 |  |  |  | 29 |  | 1 |  |  |  |  |  |  |  |  |  |  |  |  |  |  |  |  | 72 | 203 | [1] |
| **Barbados L. (BLE)** | [2] | 54 |  |  |  |  |  |  |  |  |  |  |  |  |  |  |  |  |  |  |  |  |  |  | 54 | 1504 | [1] |
| **Barbados W. (BWI)** | [2] | 3 |  |  |  |  | 6 | 21 |  |  |  |  |  |  |  |  |  |  |  |  |  |  |  |  | 30 | 150 | [1] |
| **Brazil (BRZ)** | [1] | 52 | 4 |  |  |  |  |  |  |  |  |  |  |  |  |  |  |  |  |  |  | 9 | 1 |  | 66 | 304 | [1] |
| **Costa Rica (CRI)** | [1] |  |  | 11 | 1 |  | 3 | 33 |  | 5 |  |  | 6 |  |  |  |  |  |  |  | 1 |  |  |  | 60 | 25 | [1] |
| **Cuba (CUB)** | [1] | 62 |  |  |  |  |  | 1 |  |  | 5 |  |  |  |  |  |  |  |  | 1 | 1 |  |  |  | 70 | 130 | [1] |
| **Guadeloupe (GUA)** | [1] | 2 |  |  |  |  | 71 |  |  | 1 |  |  |  |  |  |  |  |  |  |  |  |  |  |  | 74 | 151 | [1] |
| **Mexico (MEX)** | [1] |  |  |  |  |  |  |  |  |  |  |  |  |  |  | 2 | 16 | 1 | 1 |  |  |  |  |  | 20 | 311 | [1] |
| **Nicaragua (NIC)** | [1] |  |  | 19 |  |  |  | 54 | 5 |  |  |  |  |  |  |  |  |  | 16 |  |  |  |  | 1 | 95 | 205 | [1] |
| **Puerto Rico (PRV)** | [3] | 3 |  |  |  |  | 2 | 60 |  |  |  | 1 |  | 34 | 6 |  |  |  | 3 |  |  |  |  |  | 109 | 740 | [1] |
| **US Virgin Isl. (USV)** | [1] | 8 |  |  |  | 2 | 2 | 50 |  |  |  |  |  | 4 |  |  | 1 |  |  |  |  |  |  |  | 67 | 158 | [1] |
| **D.R. Jaragua (DRJ)** | PS | 1 |  |  |  |  | 2 | 3 |  |  |  |  | 1 |  |  |  | 4 |  | 4 |  |  |  |  |  | 15 | 14.6 | [4] |
| **D.R. Saona (DRS)** | PS | 3 |  |  |  |  |  | 22 |  |  |  | 2 |  | 6 |  |  |  |  |  |  |  |  |  |  | 33 | 100 | [4] |
| **TOTAL** |  | 230 | 4 | 30 | 1 | 31 | 86 | 245 | 5 | 6 | 5 | 3 | 7 | 44 | 6 | 2 | 21 | 1 | 24 | 1 | 2 | 9 | 1 | 1 | 765 |  |  |

References

1. Leroux RA, Dutton PH, Abreu-Grobois FA, Lagueux CJ, Campbell CL, et al. (2012) Re-examination of Population Structure and Phylogeography of Hawksbill Turtles in the Wider Caribbean Using Longer mtDNA Sequences. J Hered 103: 806-820.

2. Browne D, Horrocks J, Abreu-Grobois A (2010) Population subdivision in hawksbill turtles nesting on Barbados, west Indies, determined from mitochondrial DNA control region sequences. Conserv Genet 11: 1541-1546.

3. Velez-Zuazo X, Ramos WD, van Dam RP, Diez CE, Abreu-Grobois A, et al. (2008) Dispersal, recruitment and migratory behaviour in a hawksbill sea turtle aggregation. Mol Ecol 17: 839-853.

4. Revuelta O, León YM, Feliz P, Godley BJ, Raga A, et al. (2012) Protected areas host important remnants of marine turtle nesting stocks in the Dominican Republic. Oryx 46: 348-358.
